# Modern Synthetic Methods for the Stereoselective Construction of 1,3-Dienes

**DOI:** 10.3390/molecules26020249

**Published:** 2021-01-06

**Authors:** Raquel G. Soengas, Humberto Rodríguez-Solla

**Affiliations:** Department of Organic and Inorganic Chemistry, University of Oviedo, Julián Clavería 6, 33006 Oviedo, Spain

**Keywords:** 1,3-dienes, dienylation, cross-coupling, olefin metathesis

## Abstract

The 1,3-butadiene motif is widely found in many natural products and drug candidates with relevant biological activities. Moreover, dienes are important targets for synthetic chemists, due to their ability to give access to a wide range of functional group transformations, including a broad range of C-C bond-forming processes. Therefore, the stereoselective preparation of dienes have attracted much attention over the past decades, and the search for new synthetic protocols continues unabated. The aim of this review is to give an overview of the diverse methodologies that have emerged in the last decade, with a focus on the synthetic processes that meet the requirements of efficiency and sustainability of modern organic chemistry.

## 1. Introduction

### 1.1. Natural and Non-Natural 1,3-Dienes

**Figure 1:**
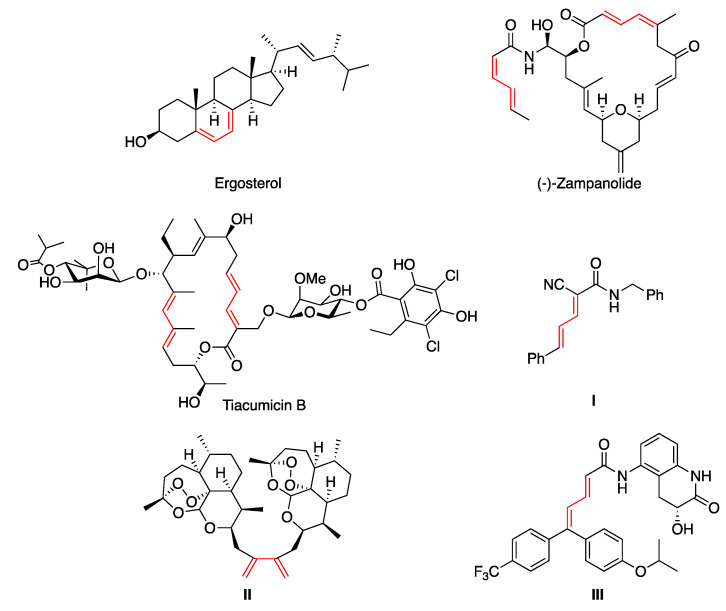
Selected examples of bioactive natural and non-natural 1,3-dienes.

The 1,3-diene structural moiety represents the key framework of many natural [1,2,3,4] and non-natural [5] products displaying a broad spectrum of biological activities [6]. Representative examples of natural products containing the 1,3-diene motif are the vitamin D2 precursor ergosterol (ergosta-5,7,22-trien-3-β-ol) [7], the potent cytotoxic agent zampanolide [8] and the macrolide antibiotic tiacumicin B [9,10] (Figure 1). Apart from natural dienes, synthetic ones have also aroused great pharmacological interest. For instance, 3-styrylacrylonitrile **I** exhibited high inhibitory potency against myeloid cell leukemia sequence 1 (Mcl-1) [11], diene dimer **II** is a potent antimalarial [12] and 5,5-diarylpentadienamide **III** is a transient receptor potential vanilloid antagonist I (TRPVI), of interest for the treatment of neuropathic pain [13] (Figure 1).

### 1.2. Applications of 1,3-Dienes

The 1,3-diene moiety have proved its usefulness and versatility in modern organic synthesis, serving as building block for the construction of important target molecules [14,15]. The reactivity of 1,3-dienes has been extensively studied, including transformations such as polymerization reactions [16,17,18], conjugate additions [19], asymmetric hydrofunctionalizations [20,21,22,23], difunctionalizations [24,25,26,27], C−H functionalizations [28,29,30,31], cycloadditions [32,33,34] and cross coupling reactions [35,36].

**Figure 2:**
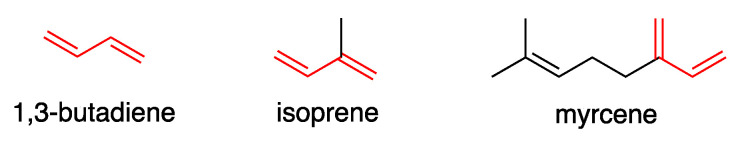
Industrially relevant 1,3-dienes.

1,3-Dienes are also important industrially. Buta-1,3-diene, produced from steam crackers on a scale of more than 10 million tons per year worldwide, is a monomer in the production of synthetic rubber. [37] Other 1,3-dienes, such as isoprene and myrcene, are also produced on a multi-ton scale and used for their conversion of into more complex and useful molecules, mainly in the polymer industry [38] (Figure 2).

### 1.3. Stereoselective Synthesis of 1,3-Dienes

In view of the important biological roles of 1,3-dienes and their relevance as synthetic intermediates, it is not surprising that their synthesis have been at the forefront of Organic Synthesis practically from its inception [39].

The stereochemistry of dienes not only influence the outcome of further chemical transformations but also determine the physical and biological properties. Therefore, it is essential that any synthesis of substituted dienes be stereoselective. The stereoselective construction of the 1,3-dienes moiety have been typically achieved by transition-metal-catalysed cross-coupling reactions between pre-fuctionalized alkenyl cross-coupling partners of predefined stereochemistry [40]. Alternatively, well-known olefination methods for the stereoselective synthesis of alkenes have been extended to the synthesis of dienes. Additional approaches include olefin metathesis and rearrangements of enynes, alkynes or allenes, for example.

### 1.4. Aim of the Review

In the last ten years, the way of understanding organic chemistry has shifted dramatically. In modern organic synthesis, there is a greater focus on economic and environmental factors, such as the use of affordable and environmentally safe reagents and solvents, the recyclability of materials or the employment of unconventional more efficient energy sources. As the literature published in the last ten years attest, the preparation of 1,3-dienes is not an exception and nowadays atom economy and sustainability are aspects of central concern in most of the synthetic protocols developed. Notwithstanding this synthetic interest, no comprehensive reviews dealing with the synthesis of 1,3-dienes have appeared in the past few years and the most recent one just covers the period 2005–2010 [41]. A review on the synthesis of 1,3-dienes has been published recently, but is restricted to the synthesis of (*E,Z*)-1,3-dienes and its application in natural product synthesis [42].

The aim of this review is to describe the most relevant progress that has been made for the preparation of 1,3-dienes from 2010 up to the present, with a focus on the application of flourishing synthetic methodologies as, among others, C-H activation, photoredox catalysis and domino and multicomponent reactions. The synthetic routes have been grouped according to the way the 1,3-diene scaffold has been assembled. Thus, syntheses of 1,3-dienes occurring via cross-coupling reactions, both transition-metal-catalysed and metal-free, are discussed in Section 2 and Section 3, respectively. Methodologies for 1,3-diene preparation centred on aldehyde dienylation are covered in Section 4, while examples of olefin methathesis-based approaches are detailed in Section 5. Section 6 is dedicated to rearrangement/isomerization reactions and, finally, Section 7 contains several miscellaneous protocols.

The present review is not intended to be an exhaustive compendium of all the recent literature in diene chemistry, but a rational and systematic presentation the most significant developments in the stereoselective synthesis of 1,3-dienes published after 2010. The examples presented in each section correspond to the selection made by the authors in an attempt to provide representative cases of interesting recent methodologies.

## 2. Transition Metal-Catalysed Cross-Coupling Reactions

During the past 50 years, transition-metal-catalysed cross-couplings have revolutionized chemical science, exemplifying one of the most powerful and popular method for the formation of carbon–carbon bonds and playing a central role in the synthesis of bioactive products and synthetic building blocks. On account of the remarkable benefits for society, the 2010 Nobel Prize in Chemistry was awarded jointly to Heck, Negishi and Suzuki “for palladium-catalysed cross couplings in organic synthesis” [43].

This section is devoted to the critical analysis of the most relevant recent strategies for the construction of the 1,3-diene moiety based in transition metal-catalysed cross-coupling reactions. For this purpose, the methodologies will be broadly divided in two groups: those based on the reaction of two sp^2^ carbons (C_vinyl_–C_vinyl_ cross couplings) and those resulting from the reaction of a sp carbon and a sp^3^ carbon (C_alkynyl_–C_alkyl_ cross couplings).

### 2.1. Carbon(sp^2^)−Carbon(sp^2^) Cross-Coupling

Transition-metal-catalysed alkenyl–alkenyl coupling is one of the most widely used methods for the synthesis of dienes and polyenes. The classical methods usually involve either the coupling of two activated vinylic carbons (generally a vinyl halide and a organometallic halide, in Suzuki or Negishi type processes) or the coupling of just one activated vinylic carbon and an alkene in a Heck-type process. More recently, the transition-metal-catalysed cross-coupling with substitution of a C-H bond, rather than a halide (so called “C-H activation”) has emerged as a highly desirable alternative, since such processes usually generate less waste and the starting materials are often easily available and inexpensive.

#### 2.1.1. Cross-Coupling of Two Activated Vinylic Carbons

**Scheme 1:**
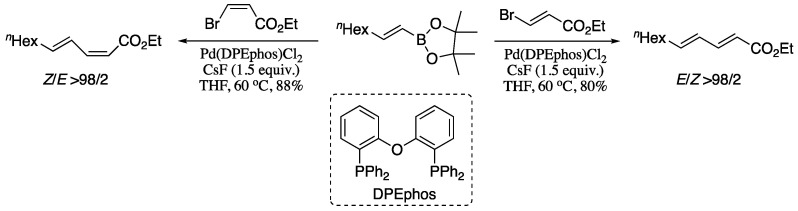
Highly selective synthesis of all four possible stereosiomers of 2,4-dienoic esters by fluoride promoted Suzuki alkenylation.

Among all the variants of the transition-metal-catalysed alkenyl–alkenyl coupling, the Pd-catalysed Suzuki-Miyaura coupling of stereodefined vinyl halides and vinylboranes reagents has long been regarded as a convenient method for the stereoselective construction of 1,3-dienes. However, stereo scrambling have been observed for several substrates. A representative example is the synthesis of dienoic esters; under Suzuki standard conditions, stereo scrambling to varying extents (≥5%) had been previously reported for the coupling of vinylboranes and 3-haloacrylates [44]. Negishi’s group found that the use of CsF as a base can suppress stereoisomerization, affording dienoic esters in good yield and excellent stereoisomeric ratio, as depicted in Scheme 1 [45].

**Scheme 2:**
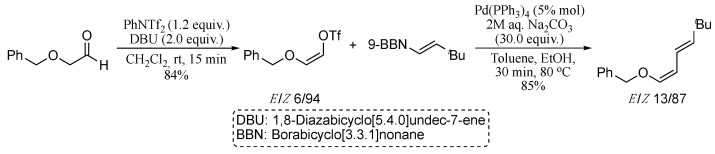
Synthesis of (*Z*)-dienes via Suzuki cross-coupling reaction.

Several recent reports describe modified Suzuki coupling procedures for the highly stereoselective synthesis 1,3-dienes. In 2015, Kurosawa and co-workers described a useful synthetic sequence for the stereoselective synthesis of diverse olefin derivatives, including 1,3-dienes, based on a (*Z*)-selective vinyl-triflation of α-alkoxyacetoaldehydes, followed by Suzuki cross-coupling (Scheme 2) [46].

**Scheme 3:**
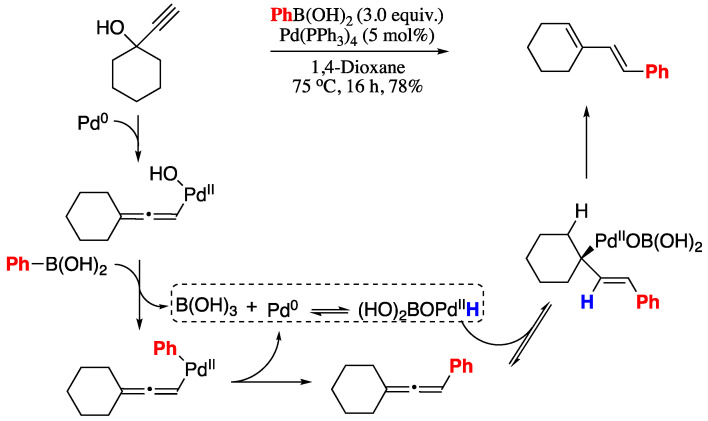
Sequential Suzuki−Miyaura coupling/palladium-mediated allene isomerization sequence.

The classical Suzuki approach for the synthesis of dienes, however, requires the hydroboration of an alkyne using air and moisture sensitive boranes to generate the corresponding vinylborane, which cannot be stored for prolonged times. A recent Suzuki-based methodology which avoids the pre-formation of unstable boranes was reported in 2016 [47]. The protocol involves a palladium-mediated, base-free, Suzuki−Miyaura coupling of propargyl alcohols and boronic acids to generate the intermediate allenes, which were then converted in situ into the corresponding 1,3-dienes by means of a hydropalladation/dehydropalladation process promoted by the formation of boric acid within the base-free Suzuki−Miyaura reaction conditions (Scheme 3).

**Scheme 4:**
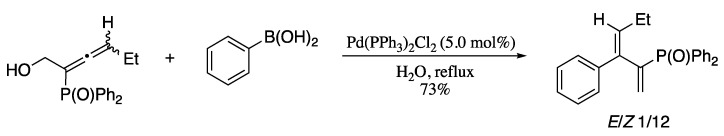
Palladium-catalysed couplings of substituted phosphinoyl-α-allenic alcohols with various arylboronic acids.

In the past few years there have been a growing interest in the use of allenic electrophiles as alternative coupling partners in Suzuki−Miyaura cross-couplings; upon reaction with a suitable boron nucleophile, coupling occurred exclusively at the central allenic carbon generating 2-substituted 1,3-dienes [48,49]. Not only this methodology give access to functionalized dienes in good yields with excellent group tolerance [50], but also have great relevance from environmental and economic points of view. For example, Liu’s group developed new routes to phosphinoyl 1,3-butadienes based on the palladium-catalysed Suzuki-Miyaura couplings of arylboronic acids with phosphinoyl-α-allenic derivatives [51] and also demonstrated that the process could be performed “on water” without surfactants or additives to afford phosphinoyl 1,3-butadienes in good yields and stereoselectivities (Scheme 4) [52].

**Scheme 5:**
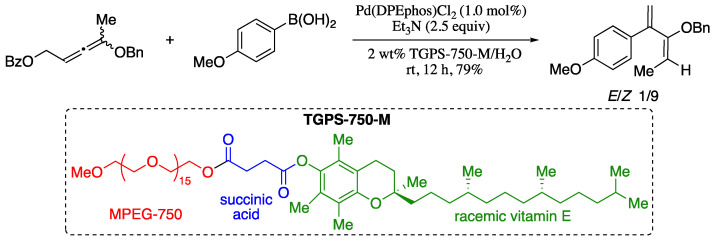
Pd-catalysed cross-couplings of substituted allenic esters in micellar medium.

As the field of green chemistry continues to expand, a number of new technologies for improving the sustainability of transition metal-catalysed processes are becoming more and more popular. In particular, switching from an organic to an aqueous micellar medium is recognized as one of the most relevant new strategies in metal-catalysis [53,54]. Following this new trend, Lipshutz and co-workers reported an environmentally responsible methodology for the preparation of substituted 1,3-butadienes based on the palladium-catalysed cross-coupling of allenes and arylboronic acids under micellar reaction conditions (Scheme 5) [55].

Despite its potential for the construction of complex dienes and polyenes, due to the strong reducing power of boranes, Suzuki reaction is subjected to severe functional group limitations. The protection with *N*-methyliminodiacetic acid (MIDA) ligand is a recently developed strategy for harnessing the reactivity of boronic acids in iterative cross-coupling (ICC) reactions aimed at the preparation of complex polyenes [56]. However, this strategy has the important drawback of the additional protection-deprotection steps.

**Scheme 6:**
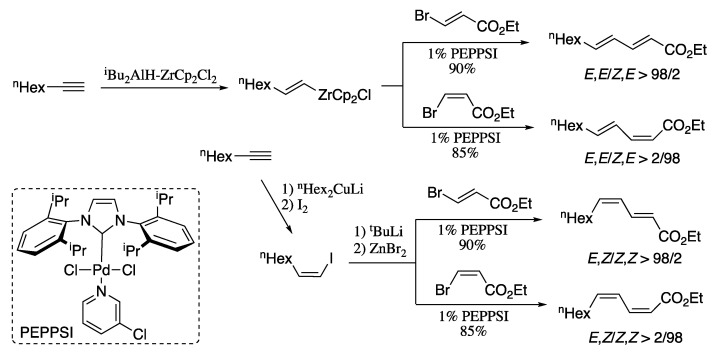
Highly selective synthesis of all four possible stereoisomers of 2,4-dienoic esters by alkyne elementometalation–Pd-catalysed Negishi coupling protocol.

As an alternative to the Suzuki cross-coupling, Negishi developed a series of strategies for the alkyne elementometalation/Pd-catalysed alkenyl–alkenyl cross-coupling tandem processes. For example, all four stereoisomers of ethyl 2,4-undecadienoate can be prepared in high selectivity by the Negishi alkenyl–alkenyl coupling (Scheme 6) [57]. On the one hand, Negishi coupling of (*E*) and (*Z*) isomers of ethyl 3-bromoacrylate with in situ generated (*E*)-1-octenylzirconocene chloride afforded (2*E*,4*E*)- and (2*Z*,4*E*)-dienes in good yields and excellent diastereoisomeric ratio. On the other hand, carbocupration of 1-octyne followed by Negishi coupling of the resulting (*Z*)-1-octenyl iodide with both (*E*) and (*Z*) isomers of ethyl 3-bromoacrylate provided isomerically pure (2*E*,4*Z*)- and (2*Z*,4*Z*)-isomers in good yields.

**Scheme 7:**
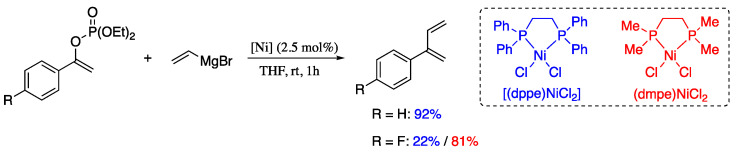
Ni-catalysed cross-coupling between vinyl phosphates and vinyl magnesium reagents.

Fiorito et al. described two nickel(II) precatalysts which enable the Kumada cross-coupling between vinyl magnesium bromide and vinyl phosphates, providing 2-substituted 1,3-dienes in good yields and with high levels of stereocontrol [58]. The cross-coupling reaction between phenyl vinylphosphate and vinyl magnesium bromide in the presence of commercial precatalyst [(dppe)NiCl_2_] afforded 2-phenyl-1,3-diene in good yield (Scheme 7). For difficult substrates such as *p*-fluorophenyl vinylphosphate, complex [(dmpe)NiCl_2_] was used as a satisfactory alternative.

**Scheme 8:**
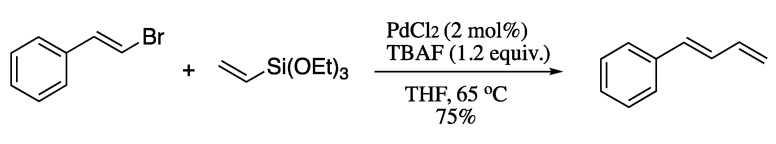
Hiyama cross-coupling catalysed by palladium nanoparticles.

Although effective, both the Negishi and Kumada processes suffer from similar problems to the Suzuki-based methodologies, as they require air and moisture sensitive conditions and in situ preparation of the vinyl intermediate. The Hiyama coupling is not subjected to these limitations, as vinylsilanes are non-toxic, stable and readily accessible. However, the classical version of the Hiyama coupling was severely limited by the requirement of a strong activator for the transmetallation step. Chatterjee et al. circumvented this issue utilizing metal nanoparticles as efficient catalysts in the presence of a mild activator such as tetrabutylammonium fluoride (TBAF) [59]. In a typical experimental procedure, styryl bromide was coupled with triethoxyvinylsilane in the presence of palladium(II) chloride and TBAF to produce the corresponding (*E*)-1,3-diene in good yields and total stereoselection (Scheme 8). The use of TBAF has a double function, as it stabilizes the Pd nanoparticles and also activates the siloxane.

**Scheme 9:**
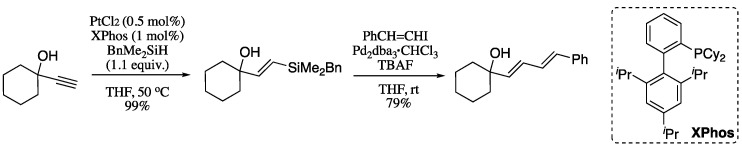
Alkyne hydrosilylation–Hiyama coupling.

Analogously to the Suzuki, Kumada and Negishi reactions, dienes can also be prepared from alkynes by the means of a hydrosilylation–Hiyama sequence. McAdam and co-workers developed an hydrosilylation methodology to form vinylsilanes from propargylic alcohols using commercially available benzyldimethylsilane; the resulting vinylsilanes are very stable and can undergo simple activation with TBAF under Trost-type conditions [60,61]. Subsequently, these authors developed a methodology for the formation of highly functionalised 1,3-dienes based on the hydrosilylation–Hiyama protocol (Scheme 9) [62]. Thus, hydrosilylation of 1-ethynylcyclohexan-1-ol afforded the corresponding vinylsilane in excellent yield and total *E* selectivity. Subsequent Hiyama coupling with iodostyrene afforded the corresponding (*E*,*E*)-diene in good yields and with total selectivity.

**Scheme 10:**
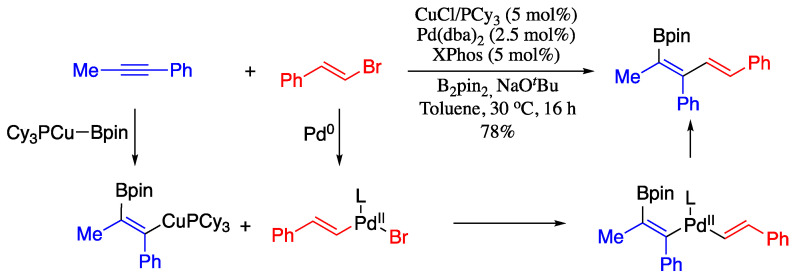
Cu/Pd-catalysed alkenylboration of alkynes.

1-Boron-1,3-dienes are important building blocks in the synthesis of a broad range of bioactive natural products and organic intermediates. In 2018, Fañanás-Mastral reported notable contribution to the stereoselective synthesis of *syn*-borylated 1,3-dienes by Cu/Pd-catalysed alkenylboration of alkynes with alkenyl bromides and bis(pinacolato)diboron [63]. This methodology involved a Cu-catalysed carboboration of alkynes to provide β-boryl-substituted alkenylcopper intermediates, which on subsequent stereoretentive Pd-catalysed cross-coupling with alkenyl halides afforded the desired *syn*-borylated 1,3-dienes (Scheme 10).

**Scheme 11:**
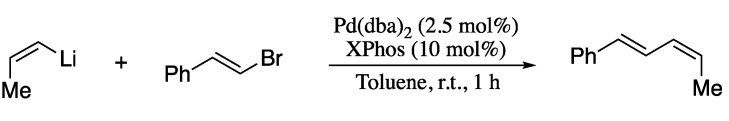
Palladium-catalysed cross-coupling of vinyllithium reagents and vinyl halides.

As seen throughout this section, organozinc, organotin, and organoboron compounds that are employed in vinyl-vinyl cross-coupling reactions, are frequently prepared from the corresponding organolithium compounds by transmetalation. Notwithstanding the evident synthetic interest, the direct cross-coupling of organolithium reagents remained virtually unexplored until very recently, due to limitations such as high reactivity and low selectivity. This has changed with Feringa’s studies on the palladium-catalysed cross-coupling of organolithium nucleophiles and organic halides [64,65,66,67]. In order to take advantage of this interesting protocol for the synthesis of 1,3-dienes, the direct cross-coupling of alkenyllithium reagents with alkenyl halides was also described (Scheme 11) [68].

**Scheme 12:**
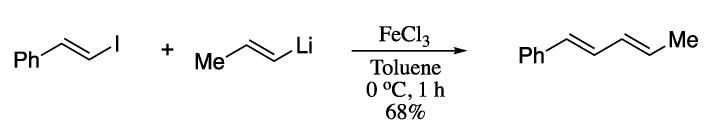
Iron-catalysed cross-coupling of (*E*)-aryl vinyl iodides with (*E*)-propenyllithium.

Following Feringa’s pioneering work on palladium-catalysed cross-coupling of alkenyllithium reagents, Liu and co-workers reported in 2018 a ligand-free iron-catalysed cross-coupling reaction of alkenyllithium compounds and vinyl iodides to form conjugated (*E*,*E*)-diene in good yields with full retention of stereochemistry (Scheme 12) [69].

**Scheme 13:**
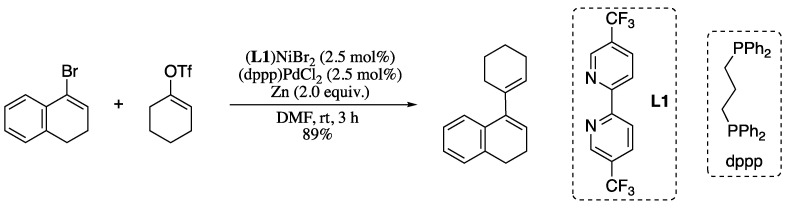
Selective cross electrophile 1,3-diene synthesis.

Even if the use of lithium organometallics is advantageous, it is obvious that the best alternative would be to avoid completely the use vinylic organometallic reagents as starting materials. In this regard, Olivares and Weix described the synthesis of 1,3-dienes by the means pf Ni/Pd cooperative catalysis in the presence of zinc as reductant to afford tetra- and penta-substituted 1,3-dienes in good yields (Scheme 13) [70].

**Scheme 14:**
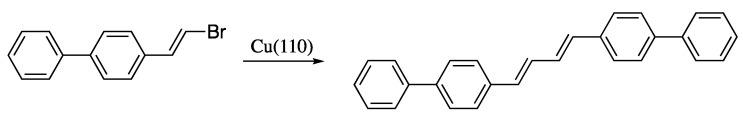
Formation of (*E*)-dienes by dehalogenative homocoupling of alkenyl bromides on Cu(110).

Yet another cross-coupling strategy for the synthesis of dienes is the dehydrogenative homocoupling of haloalkenes. A recent contribution to this field is the stereoselective synthesis of (*E*)-dienes through the dehalogenative homocoupling of alkenyl bromides on the Cu(110) surface reported by Sun et al. (Scheme 14) [71,72]. Despite the good yields and stereoselectivities and the mild conditions, this methodology is of limited interest, as it is restricted to the synthesis of symmetric dienes.

**Scheme 15:**
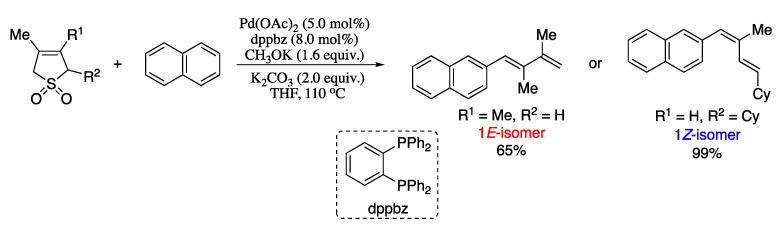
Dienylation of aromatic compounds with substituted sulfolenes.

As stated before, 1,3-dienes are typically constructed by linkage of two C=C units. However, direct attachment of the 1,3-diene moiety would allow for a more convergent and efficient strategy. Notwithstanding the evident advantages, the development of this methodology has been hampered by the lack of stable and readily available dienylation reagents. Dienylboronic acids have been explored for dienylation, but they are unstable and difficult to prepare [73]. In the search for more effective dienylation agents, Nguyen and co-workers developed a simple and practical, regio- and stereoselective dienylation employing readily available sulfolenes (Scheme 15). The regio- and stereo- selectivity are determined by the substitution pattern in the starting sulfolenes. Thus, the reaction is *E*-selective for 3,4-dimethyl sulfolene, whereas the *Z*-selective dienylation is observed for 2-cyclohexyl-4-methyl sulfolene.

**Scheme 16:**
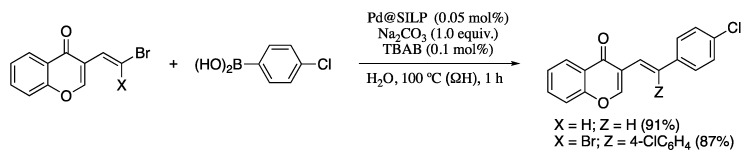
Suzuki–Miyaura reactions of chromone-based dienes with arylboronic acids.

Silva and co-corkers have recently developed an environmentally benign, economically friendly, and sustainable Suzuki–Miyaura reaction of halo and dihalo dienyl derivatives and boronic acids employing Pd(II) immobilized in a silica-supported ionic liquid in water using ohmic heating (ΩH) [74]. Ohmic heating is and advanced thermal technology recently developed for organic synthesis [75,76] which leads activation on several reactions, including transition-metal catalysed cross-couplings [77]. The combination of ohmic heating with supported ionic liquid phase catalysis (SILPC) in water offers significant advantages, as the absence of undesired side reactions, the short reaction times, the mild conditions required, the wide range of functionalities tolerated, the good yields, the environment-friendly reaction conditions, and the low catalyst loading. In addition, the supported catalyst can be recovered and maintains a good activity for at least three cycles. Thus, the Pd-catalysed coupling of bromovinyl chromones and aryl boronic acids under ohmic heating afforded the corresponding dienes in high yields and total *E* selectivity (Scheme 16). Interestingly, the coupling of 2.0 equiv. of arylboronic acids with *gem*-dibromovinyl bromoflavones afforded the corresponding bisarylvinyl chromones in good yields. For the preparation of the catalyst, Pd(OAc)_2_ was supported on amorphous silica with the aid of the imidazolium ionic liquid 1-butyl-3-methylimidazolium hexafluorophosphate ([bmim]PF_6_), which was previously physisorbed in the SiO_2_ pores.

#### 2.1.2. Cross-Coupling of One Unactivated Vinylic Carbon

The formation of C−C bonds from vinylic C−H bonds is a powerful strategy for the synthesis of dienes. Unlike the methods reviewed above, which require activation of both coupling partners, this type of processes can deliver complex and stereodefined 1,3-dienes with prior activation of just one vinylic carbon.

Among them, the most popular is the Heck vinylation reaction [78], which enables direct formation of dienes from an activated coupling partner and a vinylic C−H bond of a terminal olefin substrate [79,80]. Depending on the activated coupling partner, there are two main types of Heck vinylation reactions: the Mirozoki-Heck reaction of alkenes and vinyl halides and the oxidative Heck reaction of alkenes and vinylboronic acids.

Despite their evident synthetic potential, the classical methods for Heck vinylation has had limited application in the preparation on biologically relevant 1,3-dienes because the scope is often limited to resonance activated olefins (e.g., α,β-unsaturated carbonyls and stryenes) and also because Pd−H isomerization usually results in moderate stereoselectivity [81,82,83]. However, the past few years have witnessed important advances in the Heck vinylation reaction, that resulted in a renewed interest in the application of this methodology to the stereoselective synthesis of 1,3-dienes [84].

**Scheme 17:**
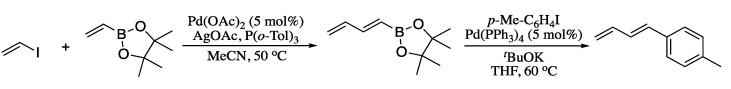
Coupling of vinyliodides with vinylboronate esters.

One those crucial recent advances in the Heck vinylation reaction was reported in 2015. In this work, Madden and co-workers demonstrated that vinyliodides could be employed as coupling partners in the Heck–Mizoroki reaction with alkenes to access directly to dienes. Interestingly, coupling of vinyliodides with vinylboronate esters afforded the corresponding dienylboronates in good yields, which can undergo a Suzuki–Miyaura coupling with a range of aryl and alkenyl halides to furnish further functionalized dienes with total *E* selectivity (Scheme 17).

It is quite surprising that, until Madden’s research, vinyliodides had been almost completely overlooked in such couplings, except for one report from Heck et al. in 1975 [85].

**Scheme 18:**
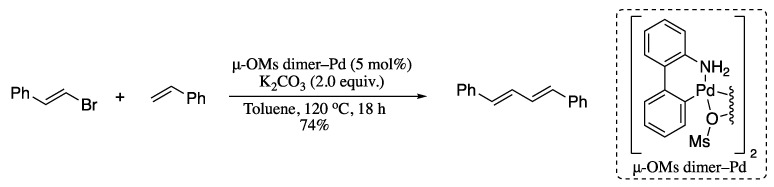
Heck reaction of alkenes and β-bromostyrenes.

The recent investigations aimed at overcoming the limitations of the Heck vinylation reaction for the construction of the 1,3-diene moiety were focused not only in the discovery of new coupling partners, but also in the development of novel catalyst systems. For example, Xu et al. developed a convenient methodology for the Heck reaction of alkenes and β-bromostyrenes based on the use of a μ-OMs palladium–dimer complex as promotor [86]. μ-OMs dimer is a non-phosphorus Pd-precatalyst which was developed by Buchwald for C–N/C–C coupling reactions [87]. When applied to the Heck reaction of (*E*)-(2-bromovinyl)benzene and styrene, the corresponding (1*E*,3*E*)-1,4-diphenylbuta-1,3-diene was isolated in good yield with total stereoselectivity (Scheme 18). It is widely assumed that the regioselectivity of the Heck vinylation reaction is substrate-controlled. Electron-deficient alkenes, such as acrylates and styrenes, afford linear dienes [88,89,90,91] whereas electron-rich alkenes, such as vinyl amides, furnish branched dienes [92,93].

**Scheme 19:**
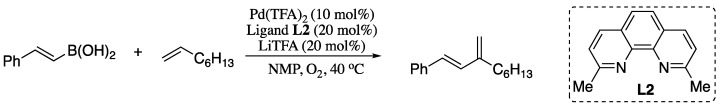
Oxidative coupling of alkenes and vinylboronic acids.

However, in 2012 Stahl and co-workers reported a oxidative coupling of alkenes and vinylboronic acids in which the regioselectivity was established via catalyst control [94]. Thus, reaction of (*E*)-styrenylboronic acid and 1-octene in the presence of palladium(II) trifluoroacetate [Pd(TFA)_2_] catalyst and neocuproine as ligand afforded the corresponding branched diene in good yield with 20/1 selectivity over the linear diene (Scheme 19).

This work represented a very significant advance in the Heck vinylation, enabling access to synthetically useful branched 1,3-disubstituted conjugated dienes and expanding the scope and synthetic utility of Heck vinylation reactions.

**Scheme 20:**
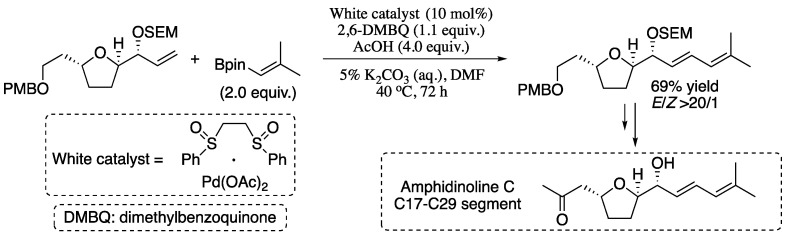
Oxidative Heck vinylation of terminal olefins and vinyl boronic esters.

In 2013, Delcamp et al. reported an oxidative Heck vinylation for the formation of dienes and polyenes [95]. In this methodology, the reaction of limiting quantities of non-stabilized terminal olefins and slight excesses of vinyl boronic esters proceeds via oxidative Pd(II)/sulfoxide catalysis to afford 1,3-dienes in good yields and excellent stereoselectivities. The potential of this powerful cross-coupling reaction in the synthesis of medicinally relevant complex diene targets was also explored. As a representative example, the synthesis of the amphidinolide C C17−C29 segment is presented in Scheme 20.

**Scheme 21:**
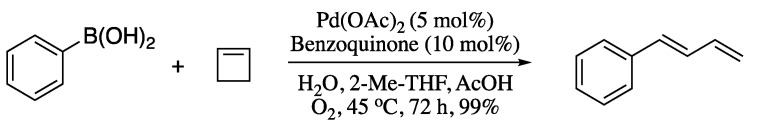
Oxidative Heck vinylation of cyclobutene with arylboronic acids.

As yet another contribution to the oxidative Heck vinylation reaction, McAlpine and co-workers reported the coupling of arylboronic acids and cyclobutene to form terminal linear 1,3-dienes in near quantitative yield and total *E* stereoselectivity (Scheme 21) [96].

**Scheme 22:**
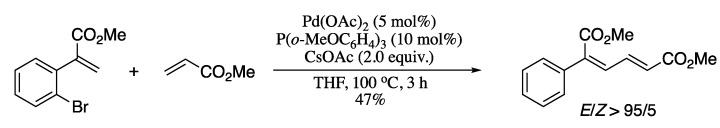
1,4-Palladium migration/Heck reaction of methyl 2-(2-bromoaryl)acrylates and methyl acrylate.

Since its discovery twenty years ago [97], 1,4-palladium migration has become an essential synthetic tool to permit the remote functionalization of C-H bonds [98,99,100,101]. Hu et al. demonstrated the usefulness of the combination of an aryl to vinyl 1,4-palladium migration with a Heck reaction for the synthesis of 1,3-dienes [102]. This method not only provides the desired dienes with high stereocontrol, but also with stereoselectivities inaccessible by previous conventional methods (Scheme 22) [103].

**Scheme 23:**
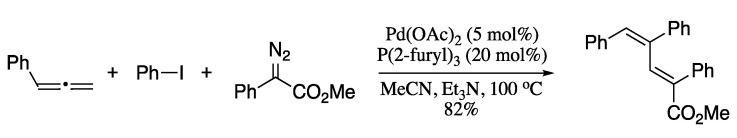
Palladium-catalysed three-component coupling of aryl iodides, allenes, and diazo compounds.

In 2013, Xiao et al. developed a novel methodology for the stereoselective synthesis of 1,3-dienes based on a three-component reaction of allenes, aryl iodides, and diazo compounds, furnishing the corresponding dienes in moderate to good yields as a single stereoisomer (Scheme 23) [104].

**Scheme 24:**
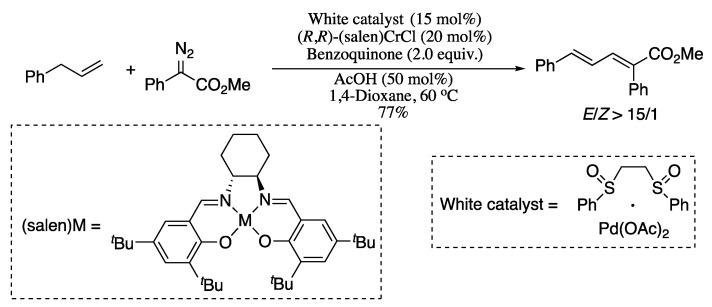
Allylic C−H olefination of α-diazo esters.

Also belonging to the subtype of transition-metal catalysed cross-coupling of one unactivated vinylic carbon is the allylic C−H olefination reported in 2014 by Wang and co-workers [105]. The olefination reaction of a wide scope of allyl compounds and α-diazo esters synergistically catalysed by a palladium(II) complex and (salen)CrCl generated diverse 1,3-diene derivatives in moderate yields and with good stereoselectivities. The (salen)CrCl was hypothesized to act as a Lewis acid to enhance the nucleophility of the α-diazo esters by coordinating to the nitrogen, thus facilitating the formation of π-allylic palladium carbenoid (Scheme 24).

**Scheme 25:**
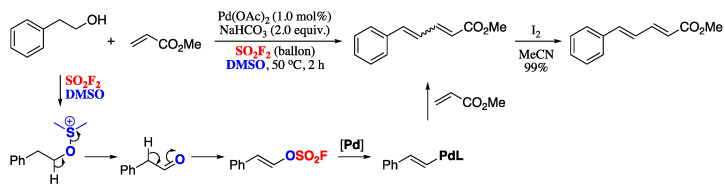
Pd-catalysed, SO_2_F_2_ mediated dehydrative cross-coupling reaction of alcohols with acrylates.

In a recent report, Revathi and co-workers developed a synthetic approach towards 1,3-dienes based on a Pd-catalysed, sulfuryl fluoride (SO_2_F_2_) mediated dehydrative cross-coupling reaction of alcohols with acrylates [106]. The process can be regarded as a Heck-type protocol, as it formally is the cross-coupling of an acrylate with an in situ formed vinyl sulfurofluoridate. Pd-catalysed reaction of an homobenzylic alcohol with methyl acrylate under a difluorosulfone atmosphere generated a mixture of *E* and *Z* dienes, which on iodine-catalysed isomerization finally furnished (*E*,*E*)-dienes in moderate yields (Scheme 25).

#### 2.1.3. Cross-Coupling of Two Unactivated Vinylic Carbons

Transition-metal-catalysed direct functionalization of C–H bonds is emerging as one of the most important tools for carbon–carbon bond formation [107,108,109,110,111,112]. In particular, direct alkenylation of an inert vinylic C−H bond is particularly attractive for constructing the 1,3-diene moiety, as alkenes are naturally abundant an readily available [113]. Moreover, a one-step conversion of C-H bonds to the desired diene functionality reduces the number of synthetic steps, thereby saving reagents, solvents and time.

In 2004, Ishii reported the direct cross-coupling reaction of vinyl carboxylate acids with acrylates [114]. Although the diene motif was effectively constructed, the yields were moderate and the stereoselectivity was poor. Despite overlooked for some years, the decade started with a renewed interest in the direct olefination of double bonds, with extensive studies by Loh [115,116,117,118], Yu [119], Ge [120], Glorious [121], Georg [122], Hong [123,124,125], Gillaizeau [126] and Liu [127,128] groups. These pioneering works have been thoroughly reviewed in 2013. Therefore, in the following part of this section, the most relevant contributions from this date will be presented.

There are two basic approaches currently being adopted for the formation of vinylic C−C bonds by combining two metal-catalysed C(alkenyl)−H activations. (a) the non-directed cross-coupling of olefins via alkenyl-Pd intermediates; (b) the transition metal-catalysed olefinic C−H alkenylation based on directed *syn* C(alkenyl)-H activation.

**Scheme 26:**
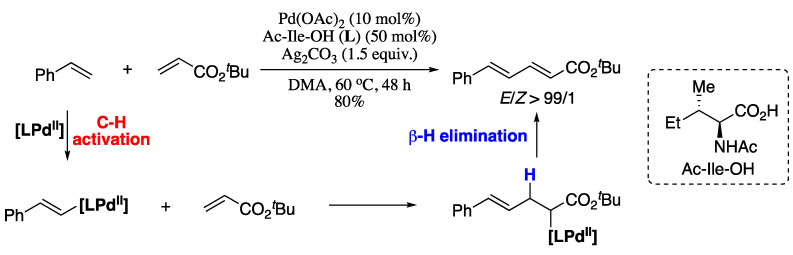
Olefination of alkenes with *tert*-butyl acrylate.

A relevant recent example of the first strategy is the palladium-catalysed cross-coupling reaction between mono-substituted common olefins and electron-deficient alkenes reported by Loh et al. in 2013 [129]. Thus, Pd-catalysed cross-coupling of alkenes with *tert*-butyl acrylate in the presence of an amino acid ligand (Ac-Ile-OH) furnished the corresponding (*E*,*E*)-diene products in good yield and excellent *E*/*Z* ratio (Scheme 26). Regarding the scope of the alkene coupling partners, various α,β-unsaturated amides, esters and phosphonates gave the corresponding butadiene products in moderate yields and good *E*/*Z* ratios.

**Scheme 27:**
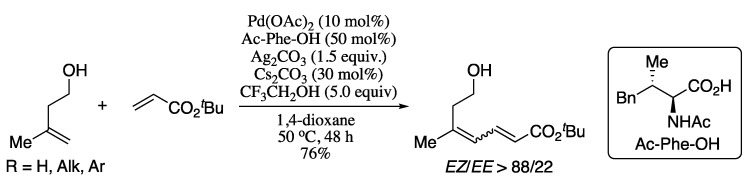
Olefination of homoallylic alcohols with *tert*-butyl acrylate.

Notwithstanding that non-directed cross-coupling was extensively studied at the beginning of the decade, this procedure is usually restricted to the synthesis of *E*,*E*-dienes. In order to switch the selectivity to the *Z*-diene, Loh’s group also developed a hydroxy group chelation-assisted procedure for the oxidative cross-coupling of alkenes and acrylates, affording *E*,*Z*-dienes in moderate yields and good *EZ*/*EE* ratios (Scheme 27) [130].

**Scheme 28:**
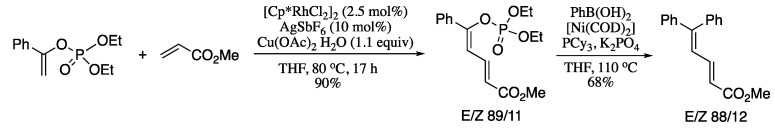
Alkenylation/Suzuki reaction of enol phosphates and acrylates.

Another significant contribution of Loh to the stereoselective C-H functionalization of alkenes utilizing directing groups was the Rh-catalysed regio- and stereoselective cross-couplings of enol phosphates with electron-deficient alkenes reported in 2015 [131]. The coupling products are also substrates for further cross-coupling reactions, giving rise to highly functionalized conjugated dienes. For example, reaction of enol phosphates with methyl acrylate in the presence of [{Cp*RhCl_2_}_2_], AgSbF_6_ and Cu(OAc) _2_·H_2_O afforded the corresponding dienol phosphate, which on Ni-catalysed Suzuki–Miyaura cross-coupling with phenyl boronic acid generated the corresponding highly functionalized diene in moderate yield and stereoselectivity (Scheme 28).

**Scheme 29:**
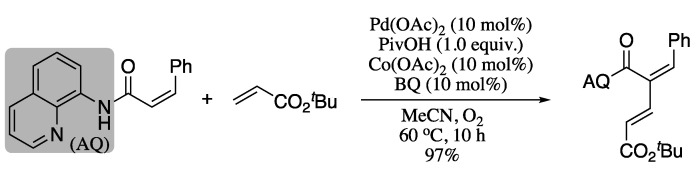
Coupling of alkene amide coupling with *tert*-butyl acrylate.

The utilization of directing groups to improve stereoselectivity in palladium-catalysed sp^2^ C-H cross-couplings via chelation was further explored by Liu et al [132]. These authors reported the synthesis of 1,3-dienes from a directing-group-containing alkene (4-pentenoic acid, allyl alcohol, or 4-pentenamine derivative) and an electron-poor coupling partner using a palladium(II)-mediated directed site-selective C(alkenyl)−H activation strategy. Reoxidation of the palladium(II) catalyst was accomplished using benzoquinone (BQ), O_2_ and catalytic Co(OAc)_2_. For example, oxidative Pd-catalysed cross-coupling of a (*Z*)-alkenamide bearing an aminoquinoline directing group with *tert*-butyl acrylate gave the corresponding diene in excellent yield and stereoselectivity (Scheme 29).

**Scheme 30:**
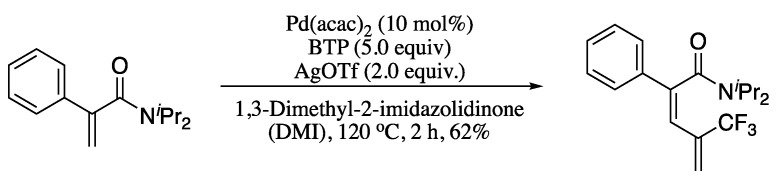
Palladium-catalysed synthesis of 3-trifluoromethyl-substituted 1,3-dienes.

As yet another relevant example of the synthesis of 1,3-dienes by means of amide-directed C−H bond functionalization, Zhao and co-workers described the palladium-catalysed cross-coupling of α-substituted acrylamides with 2-bromo-3,3,3-trifluoropropene (BTP) to produce stereoselectively trifluoromethylated 1,3-butadienes in moderate yields (Scheme 30) [133].

**Scheme 31:**
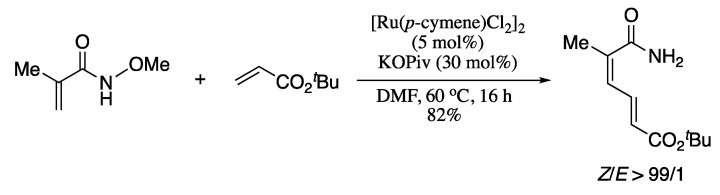
Direct cross-coupling of electron-deficient alkenes using an oxidizing directing group.

An emerging strategy in the domain of C–H activation chemistry is the use of an internal oxidizing directing group (DG^Ox^) that acts as both a directing group and an internal oxidant for redox-neutral coupling reactions [134,135,136,137]. This approach avoids the use of stoichiometric amounts of metal oxidants, improving the reaction scope on account of the milder conditions and also reducing the waste formation of the oxidant. Zhang and Zhong applied this strategy to the cross-coupling of electron-deficient alkenes, which was carried out with the assistance of the oxidizing directing group CONH(OMe) and promoted by an inexpensive ruthenium catalyst to provide 1,3-butadienes with excellent selectivities (Scheme 31) [138].

**Scheme 32:**
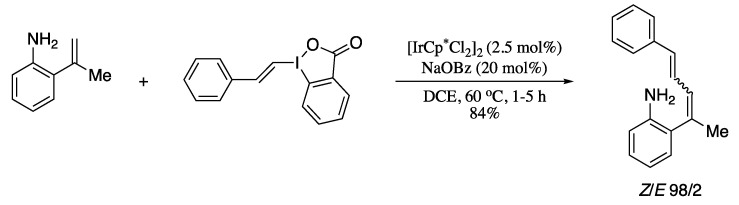
C−H Alkenylation of 2-vinylanilines with vinylbenziodoxolones.

The strategy reported by Boelke and co-workers for the directing-group-mediated C−H alkenylation also avoids the use of metal oxidants, utilizing a hypervalent iodine reagent as both oxidant and coupling partner [139]. The methodology involved aromatic amines as directing groups and alkenyl-λ^3^-iodanes as electrophilic alkene-transfer reagents, in combination with an Ir(III) catalyst (Scheme 32).

**Scheme 33:**
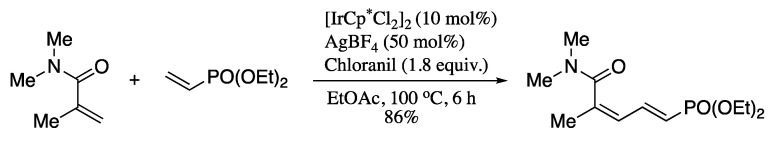
Iridium-catalysed cross-coupling reactions of alkenes.

Besides C−H alkenylation, transfer hydrogenation [140,141,142,143] is another research field of the greatest importance in modern Organic Chemistry. However, the combination of both areas remained elusive due to difficulties associated with olefin isomerization and hydrogenation of double bond. A truly game-changing report was published in 2019 and described the first example of alkene−alkene coupling integrating C−H activation and hydrogen transfer. In this work, Zhong and Zhang reported the iridium-catalysed cross-coupling between electron-deficient olefins in the presence of inexpensive chloranil as the hydrogen acceptor to provide (*Z*,*E*)-configurated dienamides [144]. Thus, reaction of acrylamides with diethyl vinylphosphonate in the presence of [IrCp*Cl_2_]_2_ (10 mol%), a silver(I) salt and chloranil as hydrogen acceptor provided stereoselectively (*Z*,*E*)-1,3-dienes in good yields (Scheme 33).

### 2.2. Carbon(sp)−Carbon(sp^3^) Cross-Coupling

Methods based on the transition metal-catalysed alkylation of alkynes can be divided into two broad types: allylic C-H functionalization and alkyne carbometallation.

#### 2.2.1. Allylic C-H Functionalization

Allylic C-H functionalization have attracted some recent interest for the synthesis of 1,3-dienes. Procedures based in allylic alkylation are redox-neutral and allow bypassing pre-functionalization of the substrates, which is very relevant from the perspective of atom economy, functional group tolerance and sustainability.

**Scheme 34:**
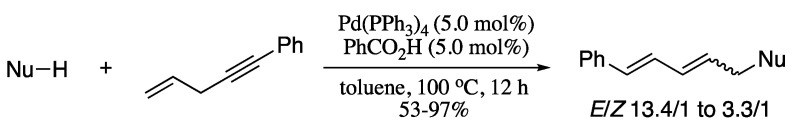
Palladium-catalysed allylic alkylation of pronucleophiles with unactivated skipped enynes.

In 2017, a seminal article was published describing the palladium-catalysed redox-neutral allylic alkylation of pronucleophiles with unactivated skipped enynes to construct 1,3-dienes with high atom economy [145]. Indolinones, cyclic ketones, diketones, esters, nitroesters, cyanoesters, carbonates and indoline and tetrahydroquinolines are all efficiently alkylated, giving the desired 1,3-dienes in moderate to good yields and moderate *E* selectivity (Scheme 34).

**Scheme 35:**
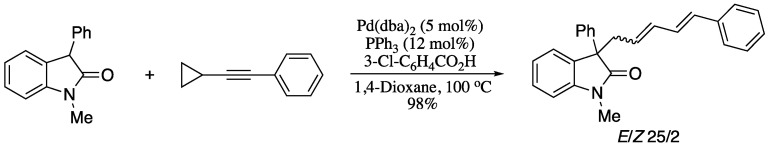
Allylic alkylation of cyclopropyl acetylene for the synthesis of 1,3-diene.

Using a similar strategy, Lu and co-workers reported the allylic alkylation of oxindole pronucleophiles with cyclopropyl aryl acetylenes [146]. Pd-catalysed cross-coupling of aromatic-substituted cyclopropylethynyls with *N*-methyl-3-phenyloxindole afforded the 1,3-diene products in good yields and moderate *E* selectivity (Scheme 35).

**Scheme 36:**
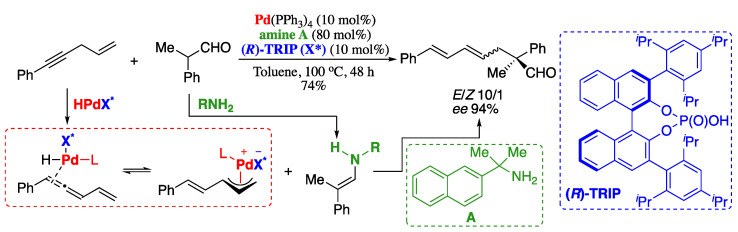
Asymmetric allylation of aldehydes with alkynes.

In 2018. Su et al. described the asymmetric α-allylation of aldehydes with alkynes promoted by a ternary catalyst system, consisting of an achiral palladium complex, a primary amine, and a chiral phosphoric acid [147]. The oxidative addition of Pd(0) to chiral phosphoric acid generated an hydridopalladium complex, which then reacted with acetylenes to form chiral electrophilic π-allylpalladium phosphate complexes. The enamine formed from the aldehyde and the amine catalyst then underwent asymmetric allylic substitution with the chiral π-allylpalladium complex, furnishing the corresponding dienes with good yields and enantioselectivities, albeit with moderate diastereoselectivity (Scheme 36).

Despite the evident synthetic potential, one source of weakness in this strategy and a problem that should be addressed in the future is the moderate stereoselectivity.

#### 2.2.2. Alkyne Carbometallation

**Scheme 37:**
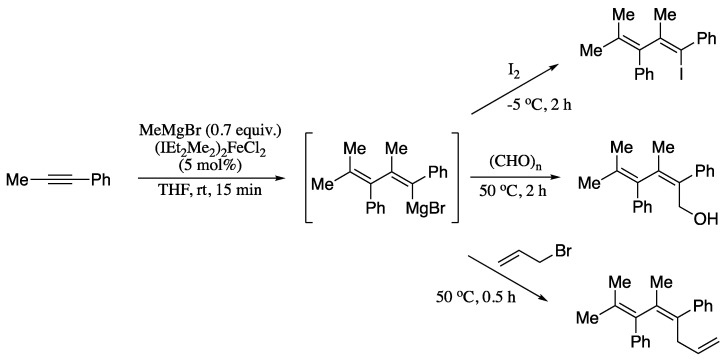
Carbometalation of phenyl(methyl)acetylene followed by transformations of the resultant 1,3-dienyl magnesium reagent.

Transition-metal-catalysed carbometalation of alkynes would be an ideal methodology for generating dienyl organometallic compounds that could be further functionalized. However, the search for a catalyst enabling such transformation proved futile until a report in 2016. In this work, Liu et al. described an iron(II)-*N*-heterocyclic carbene (NHC) complex which can serve as a precatalyst for the double carbometalation of internal unsymmetrical alkynes with alkyl Grignard reagents, producing highly substituted 1,3-dienyl magnesium reagents with high regio- and stereoselectivity (Scheme 37) [148].

The in situ formed 1,3-dienyl magnesium intermediate can also be trapped by electrophiles such as iodine, paraformaldehyde or allyl bromide to form the 1,3-dienyl iodide, the homoallylic alcohol or 1,3,6-triene, respectively, in good yields reflecting the synthetic utility of the 1,3-dienyl magnesium synthons.

## 3. Transition Metal-Free Cross-Coupling Reactions

Both the ‘classical’ transition-metal-catalysed cross-coupling methodologies based on the Heck, Negishi and Suzuki protocols and the ‘cutting-edge’ cross-coupling processes relying on the so called “C-H activation” described in the previous section have one thing in common: they are mediated by expensive late transition metals (e.g., ruthenium, rhodium, palladium and platinum). The use of these metals present two main disadvantages: on the one hand, trace metal contamination is a major problem in industry, particularly the pharmaceutical and organic electronic industries. On the other hand, the supply of these metals is at risk and it is widely assumed that their use will become unsustainable in the near future.

An important research field, therefore, involves the development of cross-coupling reactions that do not involve the use of a transition metal. There are several recent strategies for the synthesis of 1,3-dienes which are based in transition-metal free cross-coupling reactions.

**Scheme 38:**
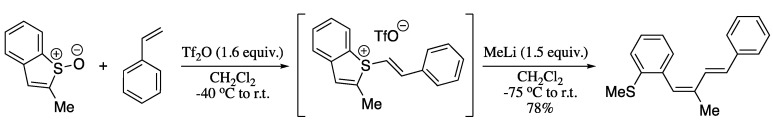
Interrupted Pummerer reaction/ligand-coupling sequence for the construction of (*E*,*Z*)-1,3-dienes.

A recent strategy to achieve the transition metal free cross coupling is the use a sulphur-based directing group to set up carbon-carbon bond formation [149]. Thus, the easy formation of a carbon-sulphur bond is used to trigger the formation of a more-challenging carbon-carbon bond in a process resembling to a reductive elimination from a transition metal, with sulphur taking the place of palladium. Such methodology was applied to the selective C(sp^2^)-C(sp^2^) coupling to deliver 1,3-dienes [150]. In this process, addition of an organolithium or organomagnesium to a key sulphurane intermediate, formed in situ by means of an interrupted Pummerer reaction of sulfoxides and alkene coupling partners, provided (*E*,*Z*)-1,3-dienes in good yields with total stereocontrol (Scheme 38).

**Scheme 39:**
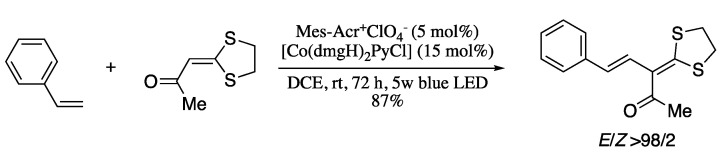
Photoinduced C(sp^2^)−H/C(sp^2^)−H cross-coupling of alkenes.

Undoubtedly, the most relevant of the emerging areas of research in synthetic chemistry is visible-light photoredox catalysis [151,152,153,154]. Photoredox catalysed cross-coupling offers an opportunity to develop greener processes and to overcome the shortcomings of using transition-metal catalysts in C−H functionalization. However, the photoredox catalysed direct C(sp^2^)−H/C(sp^2^)−H cross-coupling between two alkenes under external oxidant-free conditions was first reported in 2020. In this very recent work, Li’s group described the synthesis of substituted 1,3-dienes from vinylarenes and ketene dithioacetals under photoinduced cross-coupling reaction, providing the desired 1,3-dienes in a regio- and stereospecific manner and in good to excellent yields (Scheme 39) [155].

**Scheme 40:**
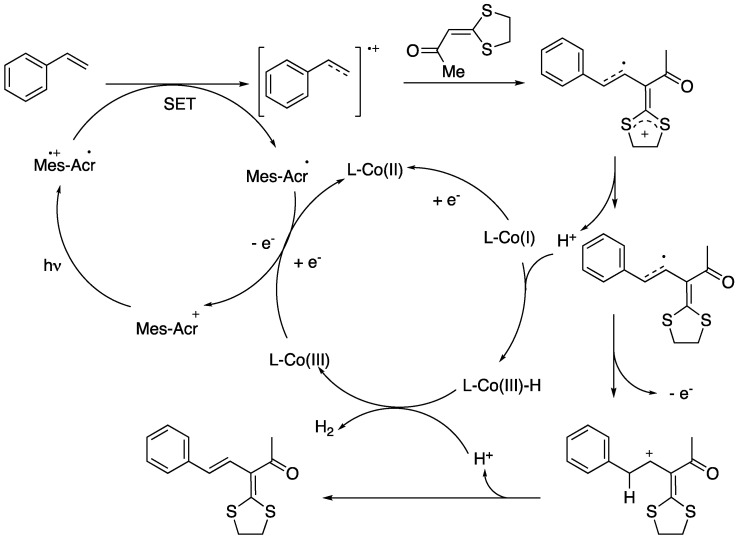
Proposed mechanism for the photoinduced synthesis of 1,3-dienes.

The proposed mechanistic cycle for the photoinduced cross-coupling reaction is depicted in Scheme 40. Initially, the styrene radical cation is formed upon electron transfer from the styrene to the excited state of the photosensitizer, generated under blue LED irradiation. Then, the nucleophilic attack of the radical cation to the α,β-unsaturated ketone would furnish a radical intermediate, which on a SET process with cobalt would form a carbocation. Finally, 1,3-diene is produced from the carbocation with the release of a proton.

It is noteworthy that the reaction can be carried out using photocatalyst and cobalt dual catalyst without using noble metal and external oxidants and with hydrogen gas as the only by-product. On view of these results and also of the great recent interest in photoredox catalysis we will, no doubt, witness more advances in the near future on the photocatalytic-based synthesis of 1,3-dienes.

## 4. Aldehyde Dienylation

**Scheme 41:**
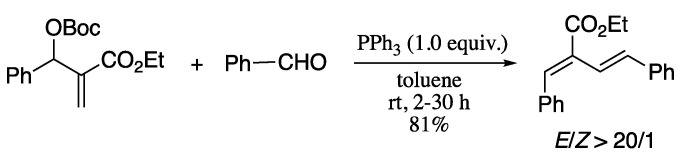
Synthesis of 1,2,4-trisubstituted 1,3-dienes from allylic carbonates and aldehydes.

For total synthesis, the ideal scenario would be the installation of the 1,3-diene moiety in one step with high stereoselectivity. In this regard, the direct synthesis of 1,3-dienes via olefination of carbonyl groups is a very attractive alternative, which have enjoyed considerable attention. Perhaps the most common strategy for the dienylation of aldehydes is the Wittig reaction and its variants. Thus, diene formation through Wittig [156,157], Wittig-Horner [158,159], Horner–Wadsworth–Emmons (HWE) [160] olefination reactions have been thoroughly investigated. However, these methodologies often require strongly basic reaction conditions and produce dienes with moderate and substrate-dependent (*E*/*Z*)-selectivity. To avoid the basic conditions and the interference from alkali metal salt by-products inherent to the conventional Wittig reaction, much effort has been directed toward developing neutral and salt-free processes. In 2010, Zhou and co-workers reported a nearly neutral and salt-free Wittig olefination between of aldehydes in the presence of allylic carbonates and a tertiary phosphine, providing a convenient methodology for the synthesis of trisubstituted 1,3-dienes (Scheme 41) [161].

**Scheme 42:**
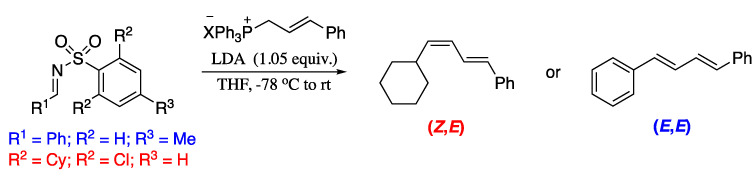
Stereoselective olefination of allylidenetriphenylphosphoranes with *N*-sulfonyl imines.

In order to improve the results of the Wittig reaction, many efforts have been devoted to the modification of semi-stabilized triphenylphosphonium ylide reagents. On contrary, little attention has been paid to the electrophiles in the Wittig reaction. In this regard, Dong et al. reported a modified Wittig dienylation protocol which consist on replacing the starting aldehyde with *N*-sulfonyl imines, which possess distinct electronic and steric properties [162]. Thus, the reaction of in situ prepared allylidene triphenylphosphoranes with a range of *p*-tolyl-activated imines provided the corresponding (*E*,*E*)-1,3-dienes, whereas the use of 2,6-dichlorobenzenesulfonyl-activated imines resulted in the exclusive formation of the (*Z*,*E*)-1,3-dienes (Scheme 42).

**Scheme 43:**
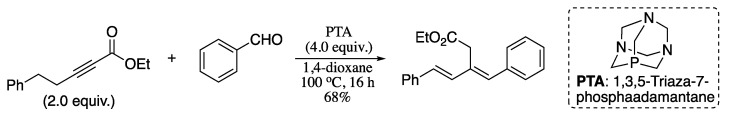
Synthesis of 1,3-dienes from aldehydes and alkynoates.

In 2011, Jacobsen and co-workers reported the Wittig olefination of phosphonium ylides generated in situ from the reaction of a 2-alkynoate and a phosphine, forming 1,3-dienes with excellent *E*-selectivity and good yields (Scheme 43) [163].

**Scheme 44:**
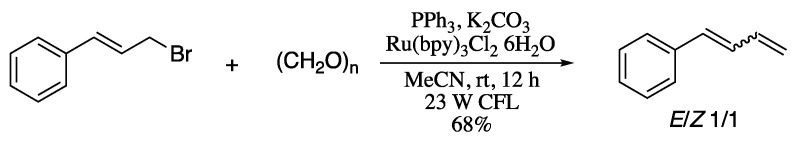
Visible-light photoredox synthesis of terminal 1,3-dienes.

As previously stated, the past few years have seen increasingly rapid advances in the field of photoredox catalysis. Inspired by the robustness of the Wittig reaction for the formation of C=C bonds and important achievements of photoredox catalysis, Fu’s group developed a methodology for the C=C bond formation via coupling of alkyl halides with aldehydes and their derivatives using triphenylphosphine as a reductive quencher, to provided terminal 1,3-dienes in good yields albeit with poor *E*/*Z* ratio (Scheme 44) [164]. This procedure enabled access to 1,3-dienes in good yields with mild reaction conditions, operational simplicity and wide functional group tolerance. An important drawback is the moderate stereoselectivity, a factor that sure will by further considered in subsequent studies. In fact, the lack of stereoselection is a recurrent problem in the Wittig-based approaches for the synthesis of 1,3-dienes.

**Scheme 45:**
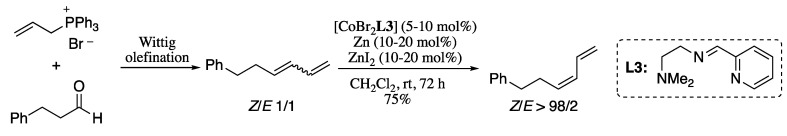
Sequence Wittig reaction/double-bond isomerization for the stereoselective generation of (Z)-dienes.

In an attempt to overcome this limitation, Hilt’s group developed a procedure for the isomerization of double bonds by applying a tridentate ligand system which, in combination with a stereo-unspecific Wittig olefination, enabled the selective formation of Z-configured 1,3-dienes (Scheme 45) [165]. In this procedure, a mixture of *E* and *Z* 1,3-dienes in a 1:1 ratio, which was generated by a stereo-unspecific Wittig olefination of octanal, is converted selectively into (*Z*)-1,3-diene in the presence of cobalt catalyst [CoBr_2_(py-imine)], zinc powder, and ZnI_2_.

**Scheme 46:**
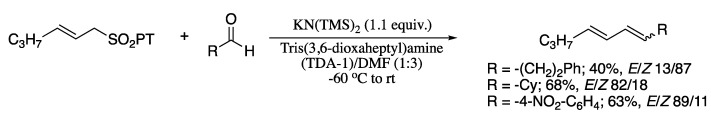
Synthesis of 1,3-dienes via Julia−Kocienski reaction.

Other classical aldehyde olefination methodology that have been widely employed for the synthesis of 1,3-dienes from aldehydes is the Julia reaction. In particular, the second-generation Julia olefination developed in mid-1990s, has become a popular synthetic method for the aldehyde dienylation on account of its wide functional group tolerance and mild reaction conditions. In general terms, the Julia–Kocienski reaction yields dienes predominantly in (*E*)-configuration on newly formed double bond. Notwithstanding, low or even inversed selectivity was also observed in several cases. In an attempt to overcome this important drawback, Billard and co-workers developed a modification of Julia−Kocienski olefination reaction based on the use of cation-specific chelating agents which provides yields 1,3-dienes with predictable (*E*/*Z*)-selectivity [166]. Thus, reaction of the potassium salt of 1-phenyl tetrazoyl (PT) sulfone with an aldehyde afforded the corresponding dienes in moderate yields (Scheme 46). Regarding the stereoselectivity, it is substrate (aldehyde) dependent. From primary α-non-branched aldehydes the (*Z*)-dienes were obtained, whereas when α disubstituted or aromatic aldehydes were used, the (*E*)-dienes were formed as main products of the reaction.

The studies presented so far provide a clear indication of the inherent limitations of the classic aldehyde olefination methodologies. Despite the recent advances in the field, none of the Wittig- and Julia-based aldehyde dienylation methods yet provided a universal solution in terms of yield and selectivity, which prompted the search for alternative procedures. Among them, processes based on a vinylogous Peterson elimination reaction have been widely investigated [167]; in fact, quite a few natural products displaying the 1,3-dienic moiety have been prepared by the means of the addition of a γ-silyl-substituted allylmetal reagent to an aldehyde followed by a Peterson-type olefination protocol [168]. For this purpose, silylated allylboronates [169], allyltitanates [170], allyl sulfonates [171] and allylzirconium reagents [172] have been used, affording dienes in high *E*-selectivity. Despite their utility, these methodologies usually lack generality and require strict reaction conditions and/or the use of highly toxic reagents. In order to overcome these limitations, a series of improved methodologies were described in the past few years.

**Scheme 47:**
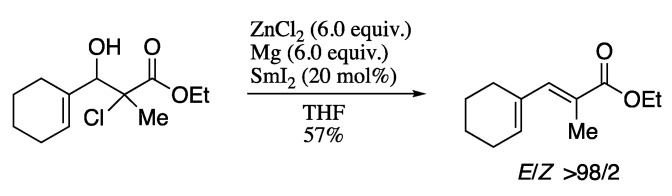
Formation of the (*E*)-dienes promoted by catalytic SmI_2_.

For example, in 2011 Rodríguez-Solla and co-workers described the synthesis of dienes with total *E*-stereoselectivity from easily available α-halo-β-hydroxy esters, with catalytic amounts of samarium diiodide in the presence of magnesium and zinc chloride (Scheme 47) [173].

**Scheme 48:**
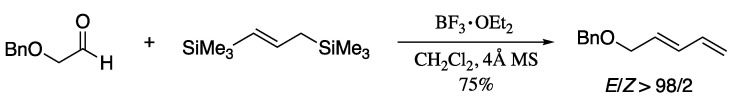
Formation of the (*E*)-dienes from aldehydes via allylsilanes.

The same year Borg et al. reported the synthesis of (*E*)-1,3-dienes via Lewis acid-promoted addition of 1,3-bis(silyl)propene to aldehydes (Scheme 48) [174].

The procedure is operationally straightforward but requires a 2.0 molar excess of a Lewis acid, which can be problematic from the point of view of functional group tolerance. Moreover, the starting 1,3-bis(silyl)propene is not commercially available and has to be prepared from relatively expensive allylsilane using highly flammable *sec*-butyllithium.

**Scheme 49:**
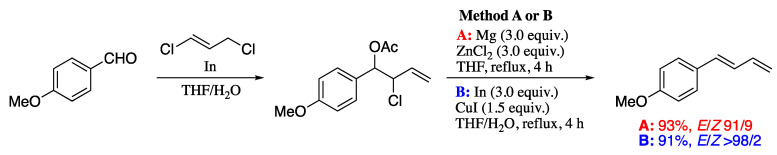
Synthesis of highly functionalized 1-substituted (*E*)-1,3-dienes by reductive β-elimination of 2-chloroallyl acetates.

As a convenient alternative, Soengas and Rodríguez-Solla developed a methodology for stereoselective synthesis of (*E*)-1,3-dienes which involves the reductive β-elimination of 2-chloroallyl acetates, prepared by indium-promoted allylation of aldehydes with commercially available and unexpensive 1,3-dichloropropene [175]. Thus, reductive β-elimination of 2-chloroallyl acetates promoted by either In/CuI or Mg/ZnCl_2_ afforded (*E*)-1-substituted-1,3-dienes in good yields with high control of the stereoselectivity (Scheme 49).

**Scheme 50:**
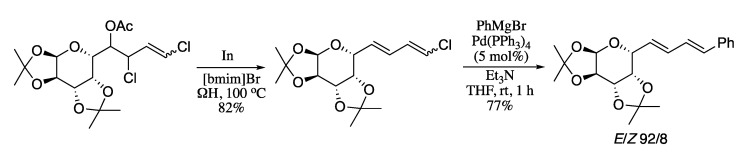
Synergic combination of ohmic heating and ionic liquids for the indium-based synthesis of 1,3-dienes.

In a further improvement of this methodology, Soengas and Rodríguez-Solla demonstrated the beneficial effects of the combination of a novel and increasingly popular reaction media such as an ionic liquid with a cutting-edge heating technology like ohmic heating (ΩH) in the dienylation reaction [176]. In this process, synthetically useful (1*E*)-4-chloro-1-substituted-1,3-dienes were prepared from 2,4-dichlorohomoallyl acetates, derived from the indium-promoted allylation of aldehydes with 3-bromo-1,3-dichloro-propene and subsequent acetylation. The elimination process was carried out by reductive β-elimination promoted by indium and, in contrast to previously reported procedures, by using ionic liquid media and ohmic heating desired 4-chloro-1,3-dienes were obtained in good yields and excellent *E*-selectivities without need of any additive. The presence of the 4-chloro-1,3-diene moiety makes these compounds ideal precursors for palladium-catalysed coupling reactions, which can be exploited for the introduction of new substituents and the elongation of the carbon chain. For example, indium-promoted allylation of a galactose-derived aldehyde, followed by acetylation, afforded the chloroacetate intermediate. Indium-promoted elimination under ohmic heating furnished the corresponding 1-chlorodiene, which on Kumada cross-coupling gave the (*E*,*E*)-diene in good yield (Scheme 50).

**Scheme 51:**
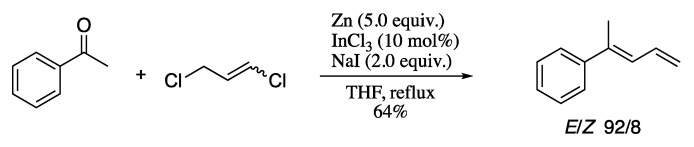
Cooperative zinc/catalytic indium system for the synthesis of highly functionalized (*E*)-1,3-dienes from carbonyl compounds.

More recently, Soengas and Rodríguez-Solla reported the stereoselective synthesis of highly functionalized (*E*)-1,3-dienes from carbonyl compounds and 1,3-dichloropropene through a cascade allylation/β-elimination reaction promoted by a cooperative zinc/catalytic indium system [177]. Thus, aldehydes or ketones were effectively dienylated in the presence of zinc and catalytic indium trichloride, affording the corresponding (*E*)-dienes in good yields and selectivities (Scheme 51).

**Scheme 52:**
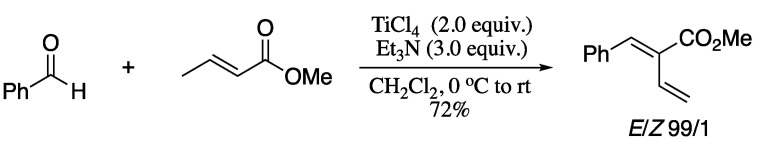
Titanium(IV) chloride-mediated stereoselective α-alkylidenation of aldehydes.

Another recent example of aldehyde dienylation is the TiCl_4_/Et_3_N-mediated condensation of aldehydes with α,β-unsaturated carboxylates to generate 1,3-dienes in good yields and stereoselectivities reported in 2016 (Scheme 52) [178]. Despite the satisfactory results, the procedure is restricted to the synthesis of butadiene-2-carboxylates.

## 5. Olefin Methathesis

**Figure 3:**
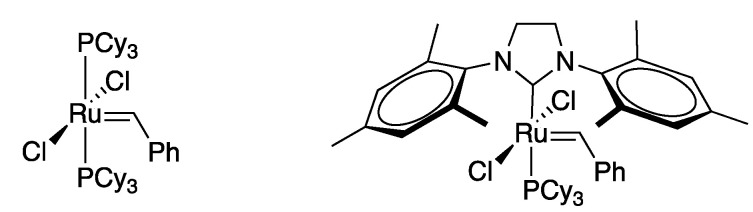
Grubbs’ catalysts.

Olefin metathesis is one of the most powerful existing methods for the formation of C-C double bonds. It basically provides the same end goal of the Wittig reaction but through a catalytic pathway. In this regard, is an extraordinarily atom-economical reaction, compatible with many functional groups. Although olefin metathesis has been known since mid-1950s, it was Grubbs’ work on ruthenium based catalysts what put this reaction at the forefront of organic synthesis [179,180] (Figure 3).

There are two basic types of olefin metathesis for the formation of C-C bonds: the cross-metathesis and the ring closing metathesis (RCM).

**Scheme 53:**
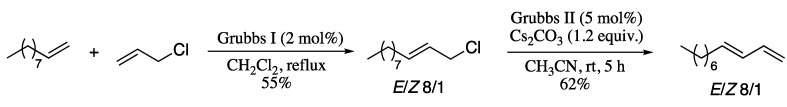
Ru-catalysed cross-metathesis/elimination sequence.

A recent example of the application of olefin cross-metathesis to the synthesis of 1,3-dienes is the cross-metathesis/elimination sequence reported by Bilel and co-workers in 2014 [181]. Thus, olefin cross-metathesis of allylic chlorides and alkenes afforded chloroallylic substrates, which on Ru-catalysed elimination generated the corresponding dienes in good yields and moderate *E*-selectivity (Scheme 53).

**Scheme 54:**
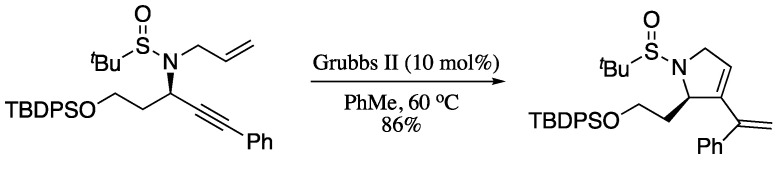
Ru-catalysed RCEYM of chiral propargyl amines.

When the ring closing metathesis is performed on an enyne system, the so-called ring closing enyne metathesis (RCEYM), it provides cyclic 1,3-dienes with one exocyclic double bond, which are of special interest for the synthesis of bioactive compounds. Both heterocyclic and carbocyclic 1,3-dienes can be prepared by means of a RCEYM reaction. For example, Tan and co-workers reported the Ru-catalysed RCEYM of chiral propargyl amines bearing an allyl group bonded to the nitrogen atom, to provide vinylpyrrolines in high yields (Scheme 54) [182].

**Scheme 55:**
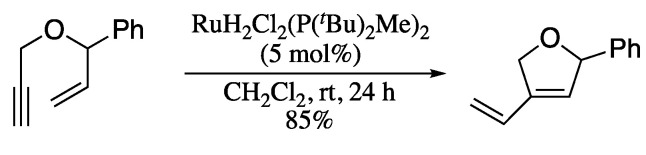
Ru-catalysed metathesis of hetero-substituted enynes.

More recently, Dolan et al. described the use of ruthenium (IV) dihydride complexes as new catalysts for the metathesis of oxygenated and nitrogenated enynes (Scheme 55) [183].

**Scheme 56:**
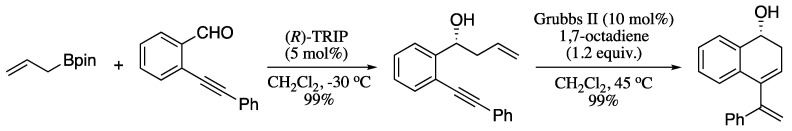
Sequential asymmetric allyl(propargyl)boration/RCEYM for the synthesis of chiral enantiopure cyclic dienes.

Some recent contributions on the synthesis of 1,3-diene carbocyclic rings includes the work of Fustero’s group on the synthesis of enantiopure cyclic dienes through the ring-closing metathesis of homoallylic benzylic or amines with an alkenyl group at *ortho*-position of the aromatic ring [184,185]. For example, chiral ω-alkynyl homoallylic(homopropargylic)alcohols, easily available from asymmetric allyl(propargyl)-boration of *ortho*-alkynyl benzaldehydes, were transformed to the corresponding cyclic 1,3-dienes via ring-closing enyne metathesis (RCEYM) (Scheme 56).

**Scheme 57:**
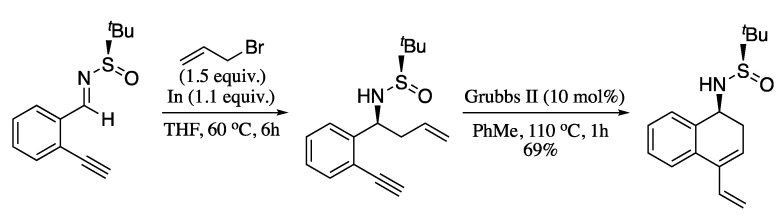
Ring-closing metathesis of enantioenriched enynes.

More recently, Yus and Foubelo performed the ring-closing metathesis of chiral *N*-*tert*-butanesulfinyl amino derivatives, prepared by means of an indium-promoted enantioselective propargylation protocol developed in their group, [186,187] producing the enantiopure cyclic dienes in high yields (Scheme 57) [188].

## 6. Rearrangement/Isomerization

Synthetic methodologies based in isomerizations or rearrangements of other chemical motifs have been extensively investigated as atom-economic alternatives for the synthesis of 1,3-dienes. Taking into account that other unsaturated functions as allenes and alkynes have the same oxidation state as 1,3-dienes, their transformation into dienes just requires some internal hydrogen reorganization. This is considerably more atom economical than external sequential reduction-oxidation operations, hence the considerable amount of literature that has been published on this matter. Rearrangements of other diene derivatives as well as other functional groups, as enols or cyclobutenes, has also been considered for the synthesis of dienes. The next section presents some major *studies* on this field that were conducted in the past decad*e*.

### 6.1. From Allenes

**Scheme 58:**
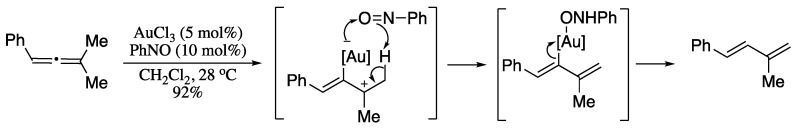
Gold-catalysed isomerization of unactivated allenes.

Translation of allene derivatives is a particularly effective method for the synthesis of functionalized dienes, thereby being a research area of much recent interest [189,190,191,192,193,194,195]. Isomerization of an allenic system to a conjugated diene was first demonstrated by Trost and co-workers [196]. Inspired by Trost’s seminal study, Tin et al. developed a gold-catalysed isomerization of unactivated allenes into 1,3-dienes [197]. Reaction of and tetrasubstituted allenes with catalytic gold(III) in the presence of nitrosobenzene as additive afforded highly functionalized dienes in excellent yields in the *E*-form only. The most likely mechanism would involve a gold allylic cation intermediate, which on an intramolecular proton transfer mediated by nitrosobenzene, generates the final 1,3-diene (Scheme 58).

**Scheme 59:**
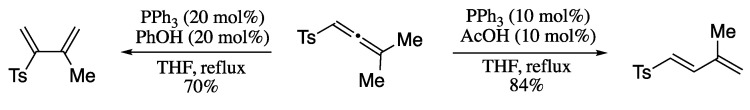
Regiodivergent synthesis of 1- and 2-arylsulfonyl 1,3-dienes.

In 2014, Hampton and Harmata reported the isomerization of allenic sulfones to arylsulfonyl 1,3-dienes [198]. Under conditions of palladium catalysis in the presence of a weak acid, allenic sulfones were converted to 1-arylsulfonyl 1,3-dienes in good yields and total stereo- and regio-control (Scheme 59).

On the other hand, nucleophilic catalysis using triphenylphosphine in the presence of a proton shuttle yields 2-arylsulfonyl 1,3-dienes.

**Scheme 60:**
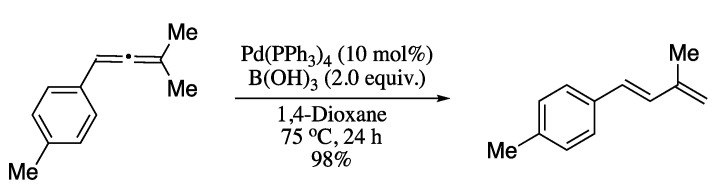
Rearrangement of unactivated allenes to 1,3-dienes.

More recently, Kimber’s group developed the rearrangement of an allene to a 1,3-diene by means of a palladium hydride complex generated in situ from a Pd(0) source and boric acid. [199] Under these conditions, allenes were transformed in 1,3-dienes in good yields and total *E*-stereoselectivity (Scheme 60). The reaction was hypothesized to occur via formation of a π-allylpalladium complex and subsequent *syn*-β-hydride elimination to give the 1,3-diene products.

**Scheme 61:**
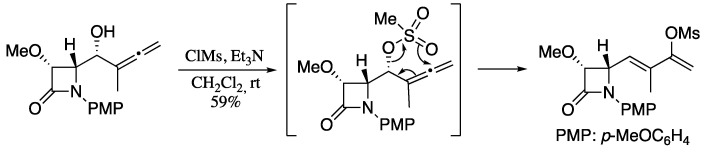
Rearrangement of allenols to 1,3-dienes.

1,3-Transposition of C-O bonds across a π-system is an important reaction in organic synthesis and has been widely studied for allylic and propargylic alcohols. On contrary, the rearrangement of allenic alcohols and their derivatives remained underdeveloped until several recent examples highlighted the interest in this transformation for the stereoselective synthesis of 1,3-dienes. In one of this pioneering works, Alcaide, observed the formation of mesylated 1,3-dienes upon treating allenic alcohols with methanesulfonyl chloride and triethylamine (Scheme 61) [200].

**Scheme 62:**
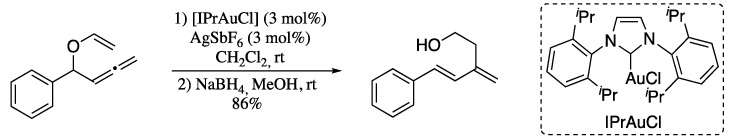
Gold (I)-catalysed Claisen rearrangement of allenyl vinyl ethers.

Later in 2011, Kraft’s group developed a gold-catalysed Claisen-type rearrangement of allenyl vinyl ethers to give 1,3-dienes with different substitution patterns (Scheme 62) [201].

**Scheme 63:**
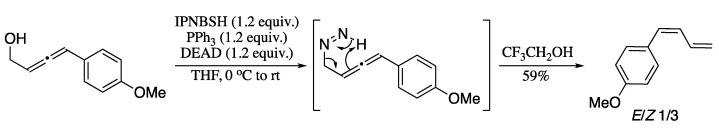
Reductive transposition of allenols.

More recently, Rinaolo et al. developed a methodology for the synthesis of 1,3-dienes via reductive transposition of allenols (Scheme 63) [202]. Reaction of 1,2-allenols with *N*-isopropylidene-*N*′-2-nitrobenzenesulfonyl hydrazine (IPNBSH) under Mitsunobu conditions [PPh_3_/diethyl azodicarboxylate(DEAD)] generated in situ the corresponding monoalkyl diazene intermediate, which upon treatment with trifluoroethanol provided through the loss of dinitrogen, 1,3-dienes in good yields albeit in moderate *Z*-selectivity.

**Scheme 64:**
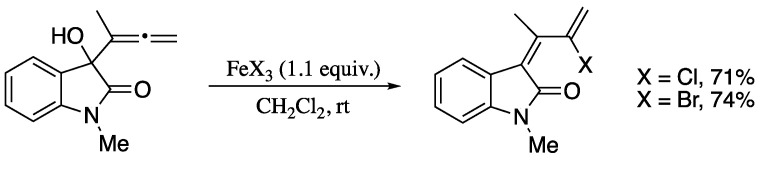
Halogenation/rearrangement of indolinone allenols.

As yet another contribution of Alcaide’s group, the synthesis of 2-halo-1,3-dienes was achieved on rearrangement of terminal allenic alcohols [203] in the presence of a stoichiometric amount of an iron(III) halide (Scheme 64) [204].

**Scheme 65:**
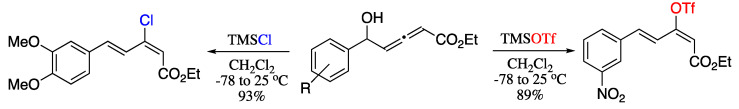
Allylic 1,3-transposition of allenols with TMSOTf or TMSCl.

In 2013, Sabbasani et al. reported a metal-free rearrangement of allenic alcohols to (*E*,*E*)-1,3-dien-2-yl triflates or chlorides by using trimethylsilyl triflate (TMSOTf) or trimethylsilyl chloride (TMSCl) respectively [205]. It was observed that the outcome of the reaction was strongly dependent on the electronic effects of the substituents. Therefore, vinyl triflates were obtained from electron-deficient substrates and TMSOTf whereas vinyl chlorides were generated on reaction of electron-rich substrates and TMSCl (Scheme 65).

**Scheme 66:**
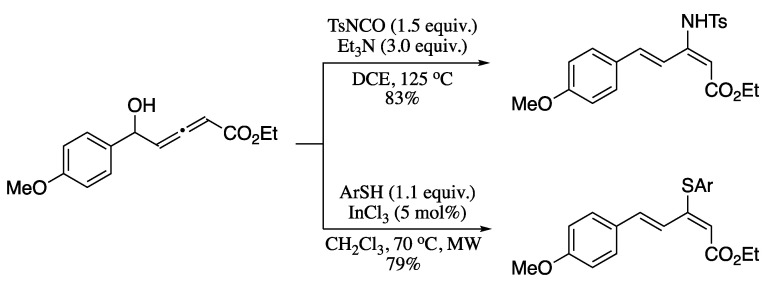
Nucleophilic additions to allenols.

Nucleophilic additions to allenols offer a unique opportunity for the synthesis of trisubstituted dienes. To date, several studies have investigated different strategies to attain this goal. For example, Wu and co-workers prepared 2-amino-1,3-dienes via metal-free decarboxylative amination of allenols by TsNCO [206], while Lee’s group described the indium(III)-catalysed addition of thiols to allenols to provide 2-thio-1,3-dienes (Scheme 66) [207].

### 6.2. From Alkynes

**Scheme 67:**
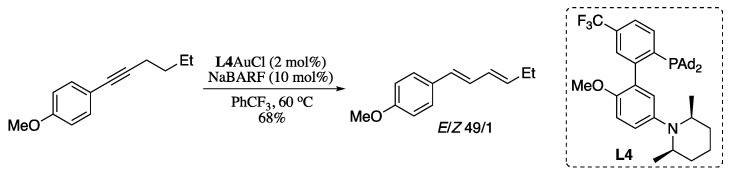
Gold-catalysed isomerization of alkynes to dienes.

Metal-catalysed alkyne isomerization has long been regarded as an efficient, atom-economical approach for the synthesis of 1,3-dienes. Several transition-metal complexes (e.g., Ru, Ir, and Pd) are known to catalyse the isomerization of acylalkynes and related substrates with strongly electron-withdrawing groups [208]. On the other hand, until recently little progress had been described for the use of unactivated alkynes as substrates [209,210,211]. During the last decade, the research on the field of alkyne isomerization was mainly focused on achieving a larger scope by developing improved strategies for the isomerization of unactivated alkynes to the corresponding 1,3-dienes. Zhang envisioned that a gold(I) complex possessing orthogonally positioned “push” and “pull” forces would enable soft propargylic deprotonation, which could led to efficient isomerization of alkynes into 1,3-dienes [212]. Thus, Zhang’s group designed a gold(I) cationic complex combining a LAu^+^ soft Lewis acid as the “pull” force with an optimally positioned basic site “pushing” force, both linked by a rigid ligand framework. Treating alkynes with the gold complex **L4**AuCl in the presence of chloride scavenger NaBARF (sodium tetrakis [3,5-bis(trifluoromethyl)phenyl]borate) resulted in the formation of the (1*E*,3*E*)-dienes in good yields and stereoselectivities (Scheme 67).

**Scheme 68:**
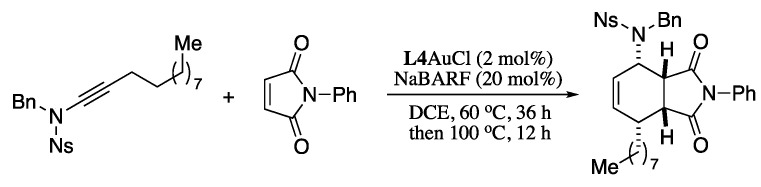
Ynamide isomerization−cycloaddition domino reaction.

In a further application of this methodology, *N*-alkynyl-*o*-nosylamides were converted to (1*E*,3*E*)-1-amido-1,3-dienes with excellent regio and stereoselectivities under gold catalysis, in a process that can be combined with a one-pot Diels−Alder reaction leading to valuable bicyclic compounds, as depicted in Scheme 68 [213].

**Scheme 69:**
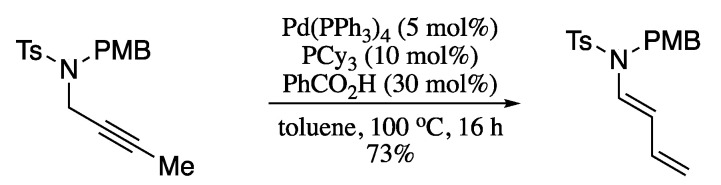
Bi-directional alkyne tandem isomerization.

In these Au-catalysed isomerizations, as well as in the classical Pd-, Ru- and Rh-catalysed reactions, the electron density of the π-system extends in a single direction. In contrast, Cera et al. disclosed in a recent report a bidirectional dual isomerization of alkynes to dienes via Pd(0)/carboxylic acid catalysis. [214] In this protocol, a palladium hydride catalyst formed in situ on combination of palladium(0) with cheap benzoic acid, enables a bi-directional p-walk involving the two α-C(sp^3^) of a 2-butynyl fragment to form the 1,3-diene moiety in moderate yields with total *E*-stereoselectivity (Scheme 69).

**Scheme 70:**
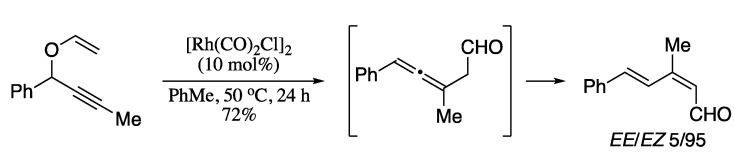
Rh(I)-catalysed tandem Claisen rearrangement/stereoselective hydrogen transfer.

The past decade has also witnessed some relevant advances in the synthesis of 1,3-dienes by means of rearrangements of propargyl alcohols. For example, Vidhani and co-workers developed a Rh(I)-catalysed approach to functionalized (*E*,*Z*)-1,3-dienals based on a tandem Claisen rearrangement/stereoselective hydrogen transfer (Scheme 70) [215]. *Z*-Stereochemistry of the first double bond suggests the involvement of a six-membered cyclic intermediate whereas the *E*-stereochemistry of the second double bond stems from the subsequent protodemetalation step giving an (*E*,*Z*)-dienal. Overall, this procedure enables access to stereodefined dienals with great atom economy and minimal waste generation.

**Scheme 71:**
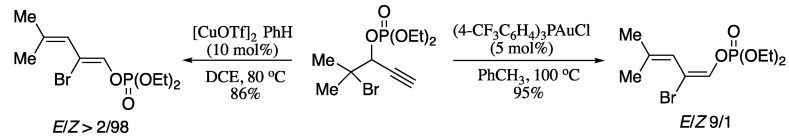
Cu-catalysed synthesis of (*Z*)-dienes and Au-catalysed synthesis of (*E*)-dienes.

In 2012, Gevorgyan and co-workers reported the synthesis of 1,3-dienes by means of a double migratory cascade reaction of α-halogen-substituted propargylic phosphates [216]. The main feature of this transformation is a 1,3-phosphatyloxy group migration followed by a 1,3-shift of an halogen, a relevant example of an extraordinarily unusual double migration of two different functionalizable groups. Moreover, this transformation is stereodivergent: copper-catalysed reactions produced (*Z*)-1,3-dienes, whereas using a gold catalyst resulted in the formation of the corresponding *E*-diene derivatives (Scheme 71).

### 6.3. From Dienes

**Scheme 72:**
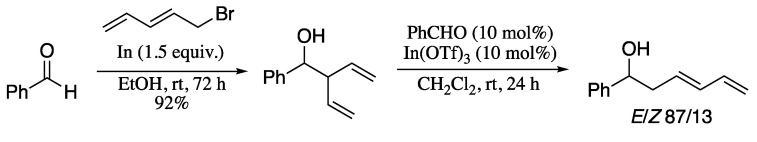
2-Oxonia Cope rearrangement of 1,4-dienols to 1,3-dienols.

1,3-Dienes can be also obtained from isomerization other dienyl derivatives. For example, it has been described that 1,4-dienols can undergo facile isomerization to the thermodynamically favored 1,3-dienols via an acid catalysed 2-oxonia-Cope rearrangement [217]. Capel et al. took advantage of this reaction to develop the synthesis of 1,3-dienols from aldehydes by means of a synthetic sequence involving the addition of pentadienylindium followed by the indium-catalysed 2-oxonia Cope rearrangement of the resulting 1,4-dienol product to the corresponding 1,3-dienol (Scheme 72) [218].

**Scheme 73:**
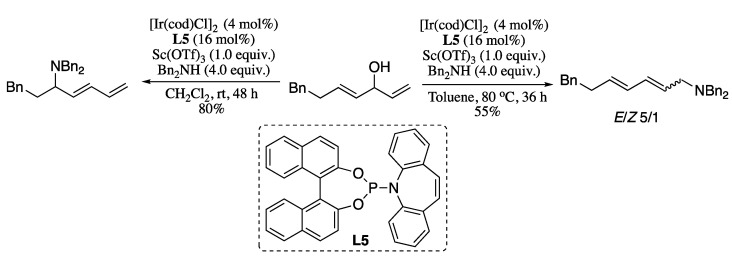
Regio-divergent allylic amination of dienyl allylic alcohols.

In a recent report, Tang et al. described an iridium-catalysed regio-divergent allylic amination of unactivated dienyl allylic alcohols [219]. The protocol proceeds under mild conditions and tolerates a wide scope of substrates, but probably the most relevant feature is the possibility of producing either branched or linear amino-1,3-dienes just by slightly tuning the reaction conditions. Thus, treatment of secondary dienyl allylic alcohols with secondary amines in the presence of an iridium catalyst and scandium triflate as additive in dichloromethane at room temperature afforded C5 amination products in good yields (Scheme 73).

On contrary, when the reaction was performed in toluene at 80 °C, the corresponding C1 amination products were obtained in moderate yields and stereoselectivities.

### 6.4. From Other Functions

**Scheme 74:**
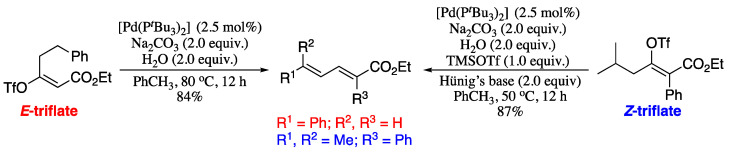
Palladium-catalysed elimination/isomerization of enol triflates.

In 2011 Crouch and co-workers reported a conceptually new route to substituted 1,3-dienes via palladium-catalysed elimination/isomerization of enol triflates [220]. Both *E*- and *Z*-enol triflates are capable of providing the corresponding functionalized, highly substituted 1,3-dienes. However, *Z*-enol triflates are considerably less reactive and require a strong Lewis acid, such as TMSOTf, to generate the corresponding dienes in synthetically useful yields. Thus, two separate sets of reaction conditions were used based on the starting olefin geometry of the enol triflate. *E*-enol triflates were treated with sodium carbonate and water at 50 °C in toluene in the presence of a palladium catalyst. Under these conditions, the corresponding (*E*,*E*)-dienes were obtained in good yields, as depicted in Scheme 74. For the *Z*-enol triflates, the above reagents and conditions were used, plus addition of trimethylsilyl triflate (TMSOTf) and Hünig’s base. Again, the (*E*,*E*)-dienes were obtained in good yields.

**Scheme 75:**
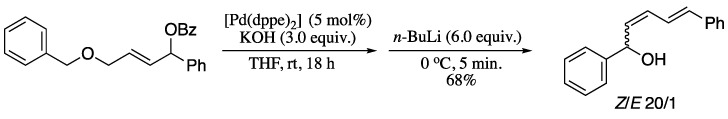
*Z*-Selective synthesis of dienyl alcohols.

Ukaji group developed the sequence 1,4-elimination/[1,2]-Wittig rearrangement for the selective synthesis of (*Z*)-dienyl alcohols. Initially, δ-benzyloxy-substituted allylic sulfones were used as starting materials, obtaining the corresponding (*Z*)-2,4- pentadien-1-ols in good yields and stereoselectivities. [221] The *Z*-selectivity was attributed to the “*syn*-effect”, namely, a stereoelectronic effect owing to σ_C−H_→π*_C=C_ interaction in the transition step of the 1,4-elimination. Notwithstanding the good results, the protocol was restricted to α,α-disubstituted allylic sulfones. When subjected to the same reaction conditions, the more acidic α-proton of α-monosubstituted allylic sulfones was deprotonated first, causing the elimination the benzyloxy group instead the sulfone moiety. In order to overcome this limitation and improve the reaction scope, Ukaji and co-workers applied the sequential 1,4-elimination and [1,2]-Wittig rearrangement δ-benzyloxy-substituted allylic benzoates, providing (2*Z*,4*E*)-2,4-pentadien-1-ols in moderate yields and stereoselectivities (Scheme 75) [222].

**Scheme 76:**
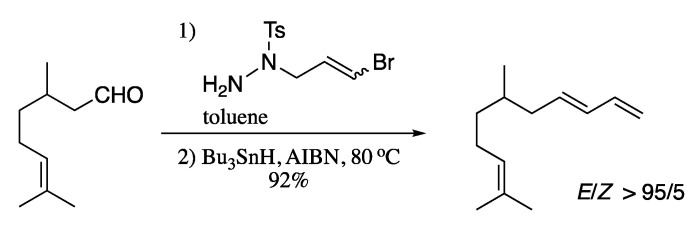
One-pot hydrazone formation followed by one-electron/pericyclic cascade for the synthesis of dienes.

Campbell and Sammis developed in 2014 a protocol for the diastereoselective synthesis of *E* dienes based on the radical cyclization of bromoallyl hydrazones [223]. This process involves a radical 6-*endo*-cyclization, followed by an elimination and a cycloreversion assisted by the release of nitrogen. Interestingly, this methodology was further extended to the direct synthesis of 1,3-dienes from aldehydes by means of a one-pot condensation/radical cyclization/cycloreversion cascade (Scheme 76).

**Scheme 77:**
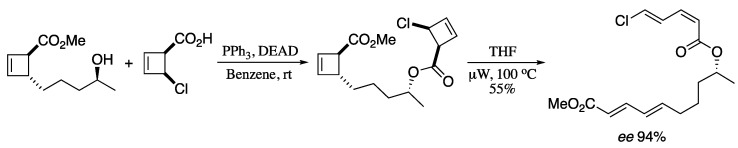
Synthesis of the southeastern fragment of Macrolactin A.

A classic example pericyclic rearrangement is the thermal conrotatory 4π-electrocyclic ring opening of cyclobutenes to afford 1,4-butadienes [224]. As the cyclobutene configuration is transferred into the diene geometry, this process generates the double bonds with high stereocontrol, hence its relevance in the context of natural products synthesis [225]. Maulide et al. decided to take advantage of the 4π-electrocyclic ring opening of cyclobutene derivatives to prepare diene carboxylates. Starting from simple bicyclic lactones, the synthesis of functionalized dienoic carboxylates was achieved by a domino allylic alkylation/4π- electrocyclic ring opening using oxygen- or nitrogen-based nucleophiles [226]. This approach has shown interesting applications in the total synthesis of diverse polyene natural products [227]. For example, a double cyclobutene electrocyclic ring opening was employed for the preparation of the southeastern fragment of macrolactin A (Scheme 77).

**Scheme 78:**
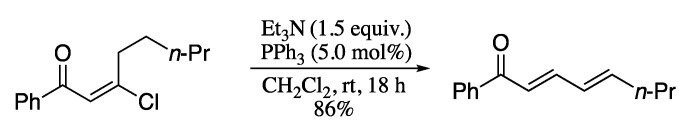
α-Vinyl enolization of (*E*)-chloroenones.

Kim and Oh extensively investigated the eliminative reaction pathways of (*E*)-β-chlorovinyl ketones and discovered that, in the presence catalytic amount of Lewis base and triphenylphosphine, 1,3-dienones were formed in high yields (Scheme 78) [228].

## 7. Miscellaneous

**Scheme 79:**
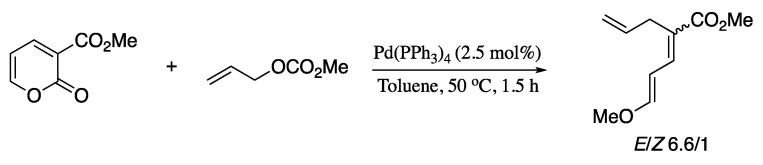
Palladium(0)-catalysed allylation of pyrones.

Maji and Tunge described an elegant palladium-catalysed decarboxylative allylation protocol for the synthesis of conjugated acyclic dienes utilizing pyrones as C4 synthons [229]. The process occurs via addition of a nucleophile generated by decarboxylation of an allylic carbonate to highly electrophilic 2-carboxypyrones (Scheme 79).

**Scheme 80:**
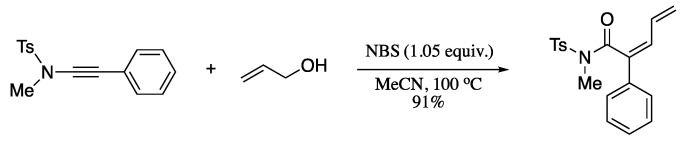
Allyloxyl addition−Claisen rearrangement−dehydrobromination cascade reaction.

Ding et al. reported a NBS-promoted allyloxyl addition−Claisen rearrangement−dehydrobromination cascade reaction of alkynylsulfonamides and allyl alcohols to provide (*Z*)-2,4-dienamides in moderate to high yields (Scheme 80) [230].

**Scheme 81:**
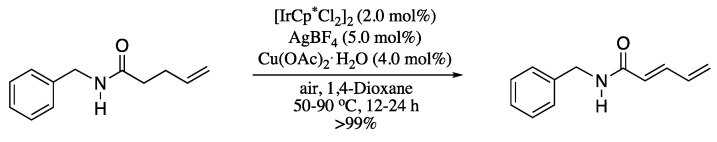
α,β-Dehydrogenation of γ,δ-unsaturated amides.

In 2018, Wang and co-workers described the preparation of conjugated dienamides and dienoic acids by the means of a direct aerobic α,β-dehydrogenation of γ,δ-unsaturated amides and acids using a simple iridium/copper relay catalysis system [231]. The reaction proceeds via allyl−Ir intermediate species, which on direct hydride elimination or ketone tautomerization, followed by 1,5-hydrogen shift, afforded the desired dehydrogenation products preserving the olefin geometry of the starting material (Scheme 81).

## 8. Conclusions

The 1,3-diene moiety has played a central role in chemical synthesis since the inception of organic chemistry as a field. This vast interest in 1,3-dienes is mainly motivated by the ubiquity of the butadiene moiety in natural and synthetic compounds of pharmacological relevance, by the emergence of diene feedstock for applications in industry and by their long-known usefulness as synthetic intermediates in a broad range of chemical processes. In this regard, the stereochemistry of dienes is crucial, since it determines the physical and biological properties but also control the outcome of further chemical transformations. Nevertheless, to date it remains challenging to target specific 1,3-diene geometries and to position substituents and functional groups along the C4 framework at prescribed locations. As it has been stated before, the synthetical interest on the stereoselective construction of the 1,3-butadienyl moiety has shown a great interest by the synthetical community since is present in many natural and non-natural products displaying a broad spectrum of biological activities. To this end, these synthetic challenges continue to fuel the interest of organic chemists in the development of novel methodologies for the stereoselective synthesis of dienes and we expect further exciting developments in coming years.

## Data Availability

Not applicable.
